# Female motivation to lead: the impact of same-sex role models and female leadership strength awareness

**DOI:** 10.3389/fpsyg.2025.1544411

**Published:** 2025-08-07

**Authors:** Sabine Boerner, Miriam Schwarzmaier, Ioannis Tagos

**Affiliations:** Chair of Organizational Behavior, University of Konstanz, Konstanz, Germany

**Keywords:** female leadership, female leadership strength awareness, gender, motivation to lead, prosocial motivation to lead, same-sex role models

## Abstract

**Introduction:**

Research on motivation to lead (MTL) suggests that women tend to be less motivated to take on leadership positions than men. By investigating female motivation to lead, we want to contest this finding.

**Methods:**

We used five samples for validating our newly specified constructs (i.e., prosocial MTL and female leadership strength awareness) in Study 1 and a further sample of 248 students in Study 2.

**Results:**

First, we propose a reconceptualization of MTL by introducing prosocial MTL as a fourth MTL type. We demonstrate that women have higher levels of prosocial MTL and non-calculative MTL, while men have higher levels of affective-identity MTL and social normative MTL. Second, we show that women are more strongly motivated to lead if they (a) have same-sex role models and (b) are aware of female strengths in leadership.

**Discussion:**

We conclude that female motivation to lead is not necessarily lower than male motivation to lead but rather different in nature, and that it can be further enhanced by factors that seem particularly relevant for women.

## Introduction

On average, only one third of leadership positions globally are held by women (World Economic Forum, 2024). While much research has investigated the roles of bias and structural discrimination in perpetuating this disparity, relatively less attention has been given to gender differences in motivational antecedents of leadership (Pillay-Naidoo and Vermeulen, 2023; Netchaeva et al., 2022). One such construct, Motivation to Lead (MTL), has been defined by Chan and Drasgow (2001, p. 482) as “an individual differences construct that affects a leader’s or leader-to-be’s decisions to assume leadership training, roles, and responsibilities and that affect his or her intensity of effort at leading and persistence as a leader.” Their model comprises three subdimensions: Affective-Identity MTL (AFF-MTL), or enjoyment and identification with leadership roles; Social-Normative MTL (SN-MTL), or a sense of obligation to lead; and Non-calculative MTL (NC-MTL), or willingness to lead despite personal costs (Badura et al., 2020).

According to role incongruity theory (Eagly and Karau, 2002), women may experience lower levels of MTL due to the mismatch between agentic traits stereotypically associated with leadership (e.g., dominance, assertiveness) and the communal traits stereotypically associated with femininity (e.g., nurturing, compassion). Although recent evidence suggests this incongruity has softened over time (Feenstra et al., 2023; Koenig et al., 2011), gender stereotypes remain a persistent barrier to women’s leadership advancement (Heilman et al., 2024). Meta-analytical evidence suggests that women tend to score lower on AFF-MTL and SN-MTL than men, possibly due to lower leader self-identification and perceived fit with traditional leadership norms. Conversely, women often report higher levels of NC-MTL than men, reflecting a communal, service-oriented motivation to lead (Badura et al., 2020; Markus and Kitayama, 1991).

These patterns, however, may not indicate a lack of motivation to lead among women but rather a limitation in how MTL has traditionally been conceptualized and measured. Specifically, existing models may privilege agentic, self-referential motives while underrepresenting communal, prosocial motivations more commonly expressed by women (Xiao et al., 2019). This paper introduces a reconceptualization of MTL by adding a fourth dimension that captures a previously under-theorized motivational pathway—one rooted in communal, social, and other-oriented reasons for assuming leadership: *Prosocial MTL (PS-MTL)*, defined as the motivation to lead driven by a desire to benefit others and make a positive social impact. Grounded in the literature on prosocial motivation (Grant and Berry, 2011) and leadership effectiveness (Northouse, 2016; Yukl, 2012), we argue that PS-MTL captures an underappreciated yet crucial aspect of motivation to lead—particularly salient for women. Our first contribution is thus to expand and recalibrate Chan and Drasgow’s (2001) model of MTL to more fully encompass the communal and social motives that may underpin especially female leadership aspirations—and those of other leaders motivated by social contribution rather than personal gain.

Second, while MTL is often viewed as a stable trait (Chan and Drasgow, 2001), emerging evidence suggests that certain contextual and cognitive factors can enhance it. For instance, women’s MTL increases when they become more aware of gender bias in leadership (Elprana et al., 2015). We therefore examine two potential moderators of the gender-MTL relationship. First, we explore the role of same-sex role models (SSRM), which have been shown to enhance leader identification in women (Fritz and van Knippenberg, 2020). Second, we introduce a novel construct, *female leadership strength awareness* (FLSA), which reflects the recognition of evidence-based advantages in female leadership style, traits, and outcomes (Boerner, 2023; Eagly, 2007; Offermann and Foley, 2020). Our second contribution, therefore, is to investigate how SSRM and FLSA might moderate the relationship between gender and the various types of MTL.

Together, these contributions aim to offer a more comprehensive understanding of motivation to lead that covers both male and female MTL, thereby advancing both theory and practice in leadership development and diversity management.

## Prosocial MTL as a novel type of motivation to lead

Chan and Drasgow’s (2001) MTL construct was inspired by Fishbein and Ajzen’s (1975) theory of reasoned action and Triandis (1980) theory of interpersonal behavior. The authors related each of their MTL dimensions to one of three aspects of a person’s social behavior (i.e., valence, social norms, and outcome). *Affective-identity* MTL (AFF-MTL), that is, “the degree to which one enjoys leadership roles and sees oneself as a leader” (Badura et al., 2020, p. 331), refers to the valence aspect. *Social-normative* MTL refers to social norms related to taking a leadership position and covers “the degree to which one views leadership as a responsibility and duty” (Badura et al., 2020, p. 331). *Non-calculative* MTL (NC-MTL), that is, “the degree to which one views leadership opportunities positively despite potential costs and/or minimal personal benefits” (Badura et al., 2020, p. 331), refers to a person’s beliefs about the outcomes of taking a leadership position.

While many scholars have applied Chan and Drasgow’s (2001) MTL construct (e.g., Elprana et al., 2015; Porter et al., 2016, 2019), the discussion about how MTL is conceptualized and measured is still ongoing (Badura et al., 2020). Based on the leadership literature, we contribute to this discussion by extending the three-dimensional MTL construct. The MTL types suggested by Chan and Drasgow (2001) are all centered to the leader’s or leader to be’s individual perspective. This leader-related view is true for affective-identity MTL (i.e., the individual joy of leading), social-normative MTL (i.e., the individual feeling of an obligation to lead), and non-calculative MTL (i.e., the individual ‘costs’ of leading).

These aspects, while important, reflect a predominantly self-referential and individualistic lens. However, in the literature, leadership is explicitly understood as a relational construct (e.g., Uhl-Bien, 2006), aiming at influencing others in order to pursue common goals or purposes (as opposed to the leader’s individual goals; e.g., Northouse, 2016; Yukl, 2012). In our view, this other-related aspect of leadership is missing in the predominantly self-related MTL construct. We therefore suggest that the motives for taking on a leadership position should be extended beyond the leader’s individual perspective by explicitly including the welfare of other people (e.g., the followers).

In order to better reflect this relational (instead of individual) nature of taking on a leadership role such as doing good for others, we draw from research on prosocial motivation which is “the desire to expend effort based on a concern for helping or contributing to other people” (Grant and Berry, 2011, p. 77). Generally, persons with high levels of prosocial motivation are expected to show commitment and dedication, pursue common goals and seeking to serve the common good, helping coworkers and display high levels of cooperation (Grant and Sumanth, 2009).

The literature on prosocial enactment of power (e.g., Baumann et al., 2016; Friedrichs et al., 2023) has recently investigated the prosocial nature of motivation to lead. Prior research observes individuals—particularly in education, healthcare, and non-profit domains—who pursue leadership to empower others and drive societal impact. For instance, based on survey data from U. S. non-profit and public employees, Piatak (2016) found that higher public service motivation was partially associated with stronger career ambitions and prosocial behaviors—suggesting how leadership aspirations can stem from a desire to serve for others. Similarly, Hameduddin and Engbers (2022) conducted a systematic review and suggested that prosocial motives could be important to leadership emergence in public service and non-profit contexts. Mergel et al. (2021), using qualitative interviews, reported that IT professionals in leading roles transitioning from the private sector to government roles often did so out of a perceived opportunity to contribute to societal good, rather than personal gain. These studies highlight how the existing MTL framework—primarily focused on personal enjoyment and identification, sense of obligation, or cost–benefit logic (Badura et al., 2020)—may overlook a critical, theoretically relevant, and empirically observable form of motivation to lead. Incorporating prosocial motivation better captures the lived realities of leaders whose motivation is intrinsically other-oriented.

We thus introduce *prosocial* motivation to lead (PS-MTL) as the degree to which one views the leadership role as a chance to help and support others. PS-MTL, that is, making a positive difference in other people’s lives by taking a leadership role, refers to the intention to contribute to the welfare of others. In order to cover the whole spectrum of possible motives for taking on a leadership role, we thus propose to complement Chan and Drasgow’s (2001) MTL conception with PS-MTL as a fourth type.

Consequently, adding PS-MTL is intended to enhance the conceptual completeness of MTL theory, making it more inclusive, ecologically valid, and socially representative (Bandalos, 2018). By integrating this prosocial dimension, we aim to account for motivational pathways that are not only prominent among women but also resonate with leaders in, for example, non-profit, healthcare, and education sectors—regardless of gender.

## Differences between female and male MTL

According to Role Incongruity Theory (Eagly and Karau, 2002), gender stereotypes may play a decisive role for the motivation to take on leadership positions (Badura et al., 2018). In their meta-analysis, Badura et al. (2020) hypothesized that AFF-MTL will be more positively related to agentic characteristics (e.g., extraversion, leader self-efficacy, narcissism) than SN-MTL and NC-MTL, while the latter will be more positively related to communal characteristics (e.g., agreeableness, horizontal and vertical collectivism) than AFF-MTL. Investigating several agentic and communal characteristics, they found partial support for their hypotheses. Moreover, they found small gender differences in that women have lower levels of AFF-MTL and SN-MTL, but higher levels of NC-MTL than men. Based on their findings, we outline our argumentation for gender differences in MTL (i.e., AFF-MTL, SN-MTL, NC-MTL, and PS-MTL).

Due to their stronger communal orientation, women (as compared to men) are more likely to perceive an incongruity between their gender role on the one hand and the agentic leadership role on the other hand (Eagly and Karau, 2002). This incongruity will negatively affect the valence aspect of their MTL (i.e., AFF-MTL). In contrast, men are likely to find their gender role and the agentic leadership role to be a good match. Men are thus more likely than women to see themselves as leaders and to enjoy the leadership role. In line with Badura et al. (2020), we thus assume that men will have higher levels of AFF-MTL than women.

Social-normative MTL refers to social norms related to taking on a leadership position (e.g., “I have been taught that I should always volunteer to lead others if I can”; Chan and Drasgow, 2001). Given the incongruence between the communal gender stereotype and the agentic leader stereotype, women are less likely to feel that taking on a leadership role is expected of them as their individual responsibility and duty. Instead, female leaders are likely to experience a so-called backlash effect in leadership (William and Tiedens, 2016): Albeit successful in their leadership role, women may be not accepted if they violate the communal gender stereotype in the eyes of others. In contrast, due to the match between their gender role and the agentic leadership role, men are more likely to feel that taking on a leadership role is their individual responsibility and duty. We therefore expect that men will have higher levels of SN-MTL than women.

Female leaders are likely to have a relational self-construal, “that is, a conception of themselves as relatively interdependent, relational, and interconnected” (Post, 2015, p. 1155), whereas as men’s self-construal is more independent (Gabriel and Gardner, 1999). Accordingly, female leaders are found to exhibit more emotional and social competence, and show more concern and empathy for their subordinates (Boerner, 2023; Post, 2015). We thus expect that female leaders (as compared to male leaders) are more likely to accept a leadership role out of selflessness rather than enjoyment (Badura et al., 2020). Therefore, they are more likely to accept personal costs when taking on a leadership role. For example, for many female leaders, the costs in terms of family care work are still higher than for male leaders (Devnew et al., 2018; World Economic Forum, 2024). In line with Badura et al. (2020), we therefore expect that women will have higher levels of NC-MTL than men.

Due to their stronger communal orientation, women generally tend to exhibit higher levels of prosocial behavior than men (Xiao et al., 2019). In their work on sex differences in emergent leadership, Eagly and Karau (1991) argue that women tend to specialize more than men in socially facilitative behaviors, while men tend to specialize more than women in behaviors strictly oriented to their group’s task. In particular, “women should engage more than men do in the socially oriented aspects of interaction and be concerned about others’ feelings and group harmony” (Eagly and Karau, 1991, p. 686). In line with gender role theory, the authors state that “women might emerge as leaders more often because of their greater attention to group morale and positive interpersonal relations.” (Eagly and Karau, 1991, p. 687).

We therefore conclude that a communal, other-oriented focus is more prominent in women’s MTL. First, women tend to have higher levels of benevolence than men, that is, promoting the maintenance and the well-being of their own group (Schwartz and Rubel-Lifschitz, 2009). Accordingly, women are found to be better in interpersonal coordination (Badura et al., 2018); in addition, female leaders tend to apply more relational leadership styles than men (Post, 2015). Second, women tend to have higher levels of universalism than men, that is, facilitating the well-being of social entities beyond their own group (Schwartz and Rubel-Lifschitz, 2009). For example, universalism is related to social justice, equality and peace (Schwartz, 2012). In sum, we assume that female leaders will have higher levels of PS-MTL than males. This assumption resonates with the differences Singer (1989) found in major determinants for leadership aspiration, a construct related to MTL (see Appendix A). While men appreciate being in a position of power and authority and the chance to assume administrative responsibilities when taking on a leadership role, women value the chance to exercise their own leadership style and having more contacts with subordinates.

Taken together, we suggest that, on average, men are likely to have higher levels of both AFF-MTL and SN-MTL due to their agentic orientation, while women will have higher levels of both NC-MTL and PS-MTL due to their communal orientation. We thus propose the following hypothesis:

*Hypothesis 1*. Men will have higher levels of (a) AFF-MTL and (b) SN-MTL than women, while women will have higher levels of (c) NC-MTL and (d) PS-MTL than men.

## How can female MTL be enhanced?

As the meta-analysis by Badura et al. (2020, p. 340) shows, gender differences in MTL cannot be generalized across different situations. In other words, some primary studies report more AFF-MTL and more SN-MTL for women than for men; in addition, in some primary studies men report higher levels of NC-MTL than women. These results point to the fact that the level of female MTL may be dependent on boundary conditions. For example, in a study on young employees in the service sector, Porter et al. (2019) found higher levels of AFF-MTL and lower levels of NC-MTL for women than for men. Moreover, women’s MTL can be facilitated by human resource practices (such as employee assessments of pay, promotion opportunities, recognition, job design, quality of organizational communications; Porter et al., 2016). Similarly, women are more strongly motivated to lead if they hold less traditional role beliefs (Elprana et al., 2015). In order to analyze further boundary conditions promoting female MTL, we examine the potential role of same-sex role models (SSRM; see Hypothesis 2) and female leadership strength awareness (FLSA; see Hypothesis 3) as moderators of the relation between gender and MTL.

### Same-sex role models (SSRM) as a moderator

Generally, a stereotype threat refers to the “the concrete, real-time threat of being judged and treated poorly in settings where a negative stereotype about one’s group applies” (Steele et al., 2002, p. 385). In a leadership role, women may experience a stereotype threat in that their communal gender stereotype is incongruent with the current agentic leadership stereotype (Eagly, 1987), resulting in expectations and feelings of inferiority. A stereotype threat in leadership can thus reduce women’s MTL if they generally feel inferior to male leaders and if they lack a feeling of social belonging (Hoyt and Murphy, 2016). This so-called vulnerability response is especially likely in settings where women are in a minority position and exposed to stereotypically masculine items (e.g., Star Trek poster or video games; Hoyt and Murphy, 2016).

Same-sex role models (SSRM) can help to protect women from vulnerability and develop a so-called reactance response, that is, engage in counter-stereotypical behavior (Hoyt and Murphy, 2016). If female leadership role models are available, the notion of role incongruity is directly disproved, thereby reducing the stereotype threat in leadership, making a leadership role more attainable to oneself. In the literature, same-sex role models have been found as a stimulating factor for both female leadership aspiration (Fritz and van Knippenberg, 2020) and female AFF-MTL (Elprana et al., 2015). We assume that SSRM are not only able to stimulate female AFF-MTL, but all four types of female MTL discussed above. Taken together, we argue that female MTL will raise if same-sex role models are available. We therefore hypothesize:

*Hypothesis 2*. The relationship between gender and motivation to lead is moderated by same-sex role models. Women who can refer to same-sex role models will have higher levels of (a) AFF-MTL, (b) SN-MTL, (c) NC-MTL, and (d) PS-MTL than women lacking same-sex role models.

### Female leadership strength awareness (FLSA) as a moderator

According to Sealy and Singh’s (2009) criteria, a role model should meet (1) similarity, (2) relevance, and (3) attainability in order to function reliably. However, given that only one third of leadership positions is held by women (World Economic Forum, 2024), not every ambitious woman will have an adequate role model at her disposition. For example, a woman in a middle management position may not feel encouraged by a female state president, whom she perceives as “too successful” to serve as a role model. In this case, identification with the female role model is unlikely. In other words, same-sex role models can also fail to encourage women to engage in leadership positions (Hoyt and Murphy, 2016).

Alternatively to individual, exemplary same-sex role models as discussed in Hypothesis 2, we suggest that female leadership strength awareness (FLSA), that is, the notion of a general strength of female leaders, may enhance female MTL. The awareness that women, in general, are successful in leadership positions, will equally help to reduce the stereotype threat in female leadership aspirants. In turn, a reduced threat might enhance female leaders to fully utilize the so-called female leadership advantage (Rosener, 1990). This advantage has been further investigated by Eagly and Carli (2003), who found that women use the *transformational* leadership style more frequently than male leaders. The authors argue that transformational leadership includes both agentic (e.g., inspirational motivation) and communal traits (e.g., individual consideration), thereby reducing the incongruity between the agentic leadership stereotype and female gender stereotype. In addition, transformational leadership meets the requirements of contemporary leadership and has proofed to be more effective than transactional leadership, which is preferred by male leaders. In sum, women’s transformational leadership style is considered an effective “middle way” between communal and agentic behaviors (Eagly, 2007, p. 4).

In her review of female leadership, Boerner (2023) found further empirical evidence for a female leadership advantage. In particular, current meta-analytical studies suggest that, on average, women have slightly higher levels of personal traits that are associated with successful leadership (i.e., extraversion, openness for experience, agreeableness, and benevolence; Anglim et al., 2022; Schwartz and Rubel-Lifschitz, 2009), while men have higher levels of narcissism (Schmitt et al., 2017; Grijalva et al., 2015). Moreover, women’s academic and professional qualifications make them at least as suitable as men for management positions (Conger and Long, 2010; Napp and Breda, 2022; Voyer and Voyer, 2014). The meta-analysis by Shen and Joseph (2021) reveals no general differences in leadership effectiveness; however, women use democratic-participative leadership styles more often than men, while men apply abusive leadership styles more often than women (Shen and Joseph, 2021). Although the reported differences between female and male leaders are minor, they consistently support the notion of a female leadership advantage.

The most recent meta-analysis by Paustian-Underdahl et al. (2024) revealed that female leaders employ more effective leadership styles than male leaders. In addition, Post (2015) referred to women’s higher relational self-construal as compared to men and demonstrated that under high coordination requirements, teams with female leaders report more cohesion and more cooperative and participative interaction norms than those with male leaders. Moreover, insights into research on gender diversity reveals that women in top management contribute to companies assuming more social responsibility (Byron and Post, 2016; Post and Byron, 2015; Velte, 2019; Wu et al., 2021).

We employed these findings on the female leadership advantage to develop the construct of female leadership strength awareness. We define *female leadership strength awareness* (FLSA) as an individual-level construct, referring to the belief in the unique strength of female leaders regarding their traits, style, and outcomes. Women’s awareness that they are generally strong and successful in leadership positions will reduce the perceived incongruity between their gender role and individual leader roles, thereby making the stereotype threat less likely. Women who are aware of the FLSA will thus develop higher levels of MTL than women who are not aware of the FLSA. In other words, we expect FLSA to operate as a moderator of the relationship between gender and MTL and hypothesize:

*Hypothesis 3*. The relationship between gender and motivation to lead is moderated by female leadership strength awareness. Women with high levels of female leadership strength awareness will have higher levels of (a) AFF-MTL, (b) SN-MTL, (c) NC-MTL, and (d) PS-MTL than women with low levels of female leadership strength awareness.

## Materials and methods

### Study 1: validation of PS-MTL and FLSA

Before testing our hypotheses in the main study (see Study 2), we validated our newly developed scales for both PS-MTL and FLSA by using five independent samples (i.e., Sample 1 to Sample 5).

*Motivation to lead (MTL)* was measured according to the scales provided by Chan and Drasgow (2001). Whereas these authors suggested MTL to be a three-dimensional second-order construct, subsequent research revealed inconsistencies in the measurement, suggesting to operationalize MTL “as three separate motivational constructs instead of as one overarching construct” (Badura et al., 2020; p. 331). Following this advice, in our reconceptualization, we measured MTL with four separate constructs (i.e., AFF-MTL, SN-MTL, NC-MTL, and the newly developed construct PS-MTL). Since our study was conducted in German universities, we used translation-back translation (Brislin, 1986) for the three established 7 point-scales—AFF-MTL (Cronbach’s alpha = 0.84–0.91), SN-MTL (Cronbach’s alpha = 0.65–0.75), and NC-MTL (Cronbach’s alpha = 0.80–0.84)—as originally reported by Chan and Drasgow (2001). In analogy to these scales, we developed a 9-item scale measuring individual differences in PS-MTL. PS-MTL, i.e., the intention to benefit others can be directed at the immediate followers (example item, “It is important to me to respond to the needs of my group through my lead”; see Table 1).

**Table 1:** Scale to measure prosocial motivation to lead (PS-MTL).

1. I do my best when knowing that my lead contributes to the well-being of others.
2. It is important to me to respond to the needs of my group through my lead.
3. As a leader, I would care about benefiting my group through my lead.
4. In a leadership position, I want to help others through my lead.
5. I would only agree to be a group leader if I had a positive impact on others.
6. If I see my positive influence, I want to take the lead.
7. I do not want to become a leader, even if others would benefit through my lead (R).
8. I am not interested to lead others, even if I see potential to benefit others through my lead (R).
9. As a leader, I want to have a positive impact on others.

To further assess whether the items of PS-MTL constitute a distinctive scale, a content analysis was conducted based on Krippendorff’s alpha statistic (Krippendorff, 2013) widely used to assess the extent to which different raters agree beyond what is expected by chance. Krippendorff’s alpha is computed based on the observed disagreement versus the expected disagreement. The formula adjusts for the chance agreement among coders, providing a more accurate measure of inter-coder reliability (Krippendorff, 2013). Four student raters were given the task to identify and categorize all 36 items measuring the four MTL types, with 9 items for each type. Furthermore, raters were provided with a general definition of each MTL type to establish a common knowledge about MTL. Krippendorff’s (2013) alpha yielded a value of 0.76, signifying a moderate to tentatively acceptable level of inter-rater agreement on the four MTL types. This result suggests that while there is some degree of consistency among raters, further refinement in measurement or categorization might enhance the reliability of the assessments.

To further provide a preliminary test of the construct, convergent, discriminant, and predictive validity of PS-MTL, we used three samples of *N* = 94 (Sample 1), *N* = 212 (Sample 2), and *N* = 227 individuals (Sample 3), each relying on a mix of student and employee respondents.

#### Psychometric properties – sample 1

An exploratory factor analysis (EFA) altogether with further preliminary checks of the psychometric properties of PS-MTL were conducted in Sample 1 (*N* = 94). Based on the EFA results (see Table 2), item 5, 7, and 8 of PS-MTL were removed as their loadings on a single factor did not or only barely meet the threshold of 0.50 (MacCallum et al., 1999). The reliability checks of the 6-item scale showed satisfactory results (Cronbach’s alpha = 0.86, omega total = 0.91, composite reliability = 0.86; Shrestha, 2021). Moreover, the scale showed a sufficient average variance extracted (AVE = 0.51). Furthermore, a confirmatory factor analysis revealed satisfying results for the six-item scale (CFI = 0.97; TLI = 0.95; RMSEA = 0.09; SRMR = 0.05; Brown and Moore, 2012).

**Table 2:** Exploratory factor analysis PS-MTL (sample 1).

Item	Factor 1	Uniqueness
PS01	0.81	0.35
PS02	0.64	0.59
PS03	0.85	0.28
PS04	0.68	0.53
PS05 *removed	0.52	0.73
PS06	0.71	0.50
PS07 *removed	0.42	0.82
PS08 *removed	0.37	0.86
PS09	0.60	0.64
*N* = 94.		

##### Construct, convergent, and discriminant validity – sample 2

As recommended by scale development literature (e.g., Hinkin, 1995) the construct, convergent, and discriminant validity was examined through Sample 2 (*N* = 212). To check on the conceptually assumed factor structure of PS-MTL, a confirmatory factor analysis was conducted, revealing mostly satisfactory results (CFI = 0.94; TLI = 0.91; RMSEA = 0.13; SRMR = 0.05; Brown and Moore, 2012).

To support the convergent validity, the composite reliability (CR) and AVE have to be above the thresholds of 0.7 and 0.5, respectively (Shrestha, 2021). The analysis showed satisfactory results for PS-MTL with a CR of 0.86 and an AVE of 0.51. Moreover, the scale showed a robust reliability (alpha = 0.85; omega = 0.91).

To test the discriminant validity of PS-MTL, leadership self-efficacy (Chan and Drasgow, 2001) and leadership aspiration (Singer, 1991) as well as all other MTL factors were used. To measure *leadership self-efficacy*, that is, a specific form of efficacy associated with the level of confidence in the knowledge, skills, and abilities associated with leading others (McCormick, 2001), we used Chan and Drasgow’s (2001) six-item scale. Example items are “Leading others effectively is probably something I will be good at,” “I believe that leading others effectively is a skill that I can master,” and “I feel confident that I can be an effective leader in most of the groups that I work with.” Cronbach’s alpha for this scale was 0.91, omega total was 0.93.

*Leadership aspiration*, that is, “the personal interest in achieving a leadership position and the will to accept the offer to take over such a position” (Fritz and van Knippenberg, 2017, p. 1019) has first been suggested by Singer (1991). Unlike the MTL construct (Chan and Drasgow, 2001), leadership aspiration is related to constructs such as career aspiration (Hoobler et al., 2014) or managerial aspiration (Dikkers et al., 2010) and does not include the motives for accepting a leadership role. We applied the six-item leadership and achievement scale by Gray and O'Brien (2007) to measure leadership aspiration (e.g., When I am established in my career, I would like to manage other employees; When I am established in my career, I would like to train others; I hope to move up through any organization or business I work in). Cronbach’s alpha for this scale was 0.82, omega total was 0.87.

According to Appendix Table A1, leadership self-efficacy is only weakly correlated with PS-MTL (*r* = 0.29; *p* < 0.05), whereas leadership aspiration shows a slightly higher correlation with PS-MTL (*r* = 0.40; *p* < 0.05). Furthermore, PS-MTL shows weak correlations with other MTL constructs such as AFF-MTL (*r* = 0.29; *p* < 0.05) as well as SN-MTL (*r* = 0.37; *p* < 0.05) but not with NC-MTL (*r* = 0.13, ns). Furthermore, we also checked on the Fornell–Larcker criterion represented through the squared correlations below the diagonal, as well as the HTMT ratio between the variables, represented through the coefficients above the diagonal in Appendix Table A2. The Fornell–Larcker criterion for discriminant validity is fulfilled if a squared correlation between the latent and discriminant variable is smaller than their respective AVE (Rönkkö and Cho, 2022). The HTMT ratio must be below the threshold of 0.85 to indicate sufficient discriminant validity (Henseler et al., 2015). Both the Fornell–Larcker criterion as well as the HTMT ratio of our data indicate sufficient discriminant validity between all constructs and therefore confirm the discriminant validity of PS-MTL.

#### Predictive validity – sample 3

Following Badura et al. (2020), MTL is predictive of leadership effectiveness. *Leadership effectiveness* was thus used to assess the predictive validity of PS-MTL, applying the 5-item scale suggested by Vecchio and Anderson (2009). Cronbach’s alpha for this scale was 0.89, omega total was 0.92. We tested the predictive validity of our six-item scale for PS-MTL (alpha = 0.87; omega = 0.92; CFI 0.95; TLI = 0.92; RMSEA = 0.13; SRMR = 0.05) by regressing leader effectiveness on control variables (i.e., gender, age, academic degree, past leadership experience, first-generation college student, leadership self-efficacy, leadership aspiration) and the four types of MTL. We tested the predictive validity of our scale for PS-MTL by using a sample of *N* = 227 (Sample 3). Descriptive statistics and correlations for this sample are provided in Appendix Table A3.

Similar to the results of Badura et al. (2020), AFF-MTL is the best predictor for leadership effectiveness (ß = 0.39, *p* < 0.001; see Appendix Table A4). Thus, those who are motivated to lead because they enjoy the leadership role as such perceive themselves as effective leaders. While both SN-MTL and NC-MTL were unrelated to leadership effectiveness, the newly developed type of PS-MTL significantly contributes to predict leadership effectiveness (ß = 0.19, *p* < 0.001). In other words, those who are motivated to lead out of prosocial motives equally perceive themselves as effective leaders, over and above their level of AFF-MTL. Compared to Model 4, including only the three types of MTL suggested by Chan and Drasgow (2001), Model 5, complemented by PS-MTL, results in a significant rise of the explained variance in leadership effectiveness (*R*^2^ adjusted = 0.44; *p* < 0.001). Although our measure for leadership effectiveness relies on self-assessments and our data may thus suffer from common method bias, we interpret these results as a first indication for the predictive validity of our newly developed type of PS-MTL.

##### Female leadership strength awareness (FLSA)

Female leadership strength awareness was designed according to empirical findings on the female leadership advantage (Boerner, 2023). In order to assess the participant’s awareness of the female leadership advantage, they were transformed into items of a 6-item scale (Staneker, 2022). This procedure was inspired by Elprana et al. (2015) who created their scale for awareness of gender inequality (AGI) based on the Modern Sexism Scale (Eckes and Six-Materna, 1998). A sample item is “Women use more relations-oriented leadership styles (e.g., caring, democratic, participative) than men” (see Table 3). After eliminating item 2 and item 3 because of poor factor loadings (i.e., below 0.60; MacCallum et al., 1999), Cronbach’s alpha for this scale was 0.85, omega total was 0.86, CR was 0.84, and AVE was 0.59. A confirmatory factor analysis verified the one-dimensional structure of this variable (CFI = 1.00; TLI = 1.00; RMSEA = 0.03; SRMR = 0.02).

**Table 3:** Items female leadership strength awareness (FLSA).

1. Women have more advantageous leadership traits than men (such as extraversion, openness to experience, agreeableness and benevolence).
2. Women’s academic and professional qualifications make them at least as suitable as men for management positions.
3. Women are at least as successful as men in leadership positions.
4. Women in top management contribute to companies assuming more social responsibility.
5. Women use more relations-oriented leadership styles (e.g., caring, democratic, participative) than men.
6. Women lead less abusively (e.g., derogatorily, exposingly) than men.

To provide a preliminary test of the construct, convergent, discriminant and predictive validity of our newly developed scale for FLSA, we used a mix of student and employee samples of *N* = 212 (Sample 2), *N* = 295 (Sample 4), and *N* = 551 individuals (Sample 5).

#### Psychometric properties – sample 4

According to an exploratory factor analysis (see Table 4) for Sample 4 (*N* = 295), item 2 and item 3 of the scale for FLSA were removed since their factor loadings did not meet the threshold of 0.50 (MacCallum et al., 1999). The final scale consists of 4 items with a satisfactory reliability and AVE above the respective threshold of 0.7 for reliabilities and 0.5 for AVE (alpha = 0.76; omega = 0.87; CR = 0.80; AVE = 0.51; Shrestha, 2021). A confirmatory factor analysis also showed mostly satisfying results (CFI = 0.99; TLI = 0.96; RMSEA = 0.09; SRMR = 0.02; Brown and Moore, 2012).

**Table 4:** Exploratory factor analysis FLSA (sample 4).

Item	Factor 1	Uniqueness
FLSA01	0.59	0.65
FLSA02 *removed	0.16	0.97
FLSA03 *removed	0.24	0.94
FLSA04	0.74	0.45
FLSA05	0.80	0.36
FLSA06	0.70	0.51
*N* = 295		

##### Construct, convergent, and discriminant validity – sample 2

Examining Sample 2 (*N* = 212), a confirmatory factor analysis revealed satisfying results for the FLSA construct (CFI = 1.00; TLI = 1.00; RMSEA = 0.00; SRMR = 0.01) (Brown and Moore, 2012). Furthermore, to examine the convergent validity of FLSA, the construct showed a satisfactory reliability and AVE (alpha = 0.81; omega = 0.83; CR = 0.81; AVE = 0.52).

To assess the discriminant validity of FLSA, we tested a range of constructs that may be related to his variable. More precisely, we tested the correlations between FLSA and leadership self-efficacy (alpha = 0.91; omega = 0.95), leadership aspiration (alpha = 0.83; omega = 0.90), awareness of gender inequality (alpha = 0.82; omega = 0.80), and SSRM. As documented in Appendix Table B1, FLSA is only weakly correlated to any of these variables, indicating preliminary evidence for the discriminant validity of the construct. FLSA significantly correlates with leadership self-efficacy (*r* = 0.15, *p* < 0.05), leadership aspiration (*r* = 0.17, *p* < 0.05), and awareness of gender inequality (*r* = −0.26, *p* < 0.05) but not with SSRM (*r* = 0.03, ns). Interestingly, FLSA is moderately correlated with female gender (*r* = 0.40, *p* < 0.05).

Furthermore, we also checked on the Fornell–Larcker criterion, as well as the HTMT ratio between the variables (see Appendix Table B2). Both, the Fornell–Larcker criterion as well as the HTMT ratio indicate sufficient discriminant validity between the constructs and for the construct of FLSA.

#### Predictive validity – sample 5

We assume that FLSA (alpha = 0.79; omega = 0.81; CFI = 1.00; TLI = 1.00; RMSEA = 0.00; SRMR = 0.01), by countervailing the stereotype threat, will enhance both women’s MTL and their leadership effectiveness. *MTL* (i.e., AFF-MTL, SN-MTL, NC-MTL, and PS-MTL) and *leadership effectiveness* were thus used to assess the predictive validity of FLSA, controlling for age, academic degree, first-generation college student, and past leadership experience. With the exception of NC-MTL, FLSA was predictive of both MTL and leadership effectiveness, supporting the predictive validity of the newly developed variable (see Appendix Tables B3, B4).

### Study 2: test of hypotheses

#### Measures

For Study 2 and relying on a new and independent sample (*N* = 248; see below), we used Chan and Drasgow’s (2001) established MTL scales and reached sufficient reliability coefficients for AFF-MTL (alpha = 0.96; omega = 0.97), SN-MTL (alpha = 0.89; omega = 0.92), and NC-MTL (alpha = 0.89; omega = 0.91; see Table 5).

**Table 5:** Descriptive statistics and correlations among study variables.

Variables	Mean	SD	(1)	(2)	(3)	(4)	(5)	(6)	(7)	(8)	(9)	(10)	(11)
(1) Gender	0.68	0.47	–										
(2) Age	23.3	3.86	0.01	–									
(3) Academic degree	0.40	0.49	0.23*	0.43*	–								
(4) First-gen	0.28	0.45	−0.17*	0.10	−0.05	–							
(5) PLE	0.71	0.43	0.07	0.11	0.13*	−0.12	–						
(6) SSRM	0.63	0.48	0.14*	−0.13*	0.13*	−0.52*	0.23*	–					
(7) FLSA	5.18	1.25	0.48*	−0.06	0.25*	−0.23*	0.14*	0.25*	(0.85)				
(8) AFF-MTL	4.40	1.54	−0.16*	0.02	−0.20*	0.06	−0.09	−0.08	−0.10	(0.96)			
(9) SN-MTL	3.75	1.23	−0.31*	−0.05	−0.23*	0.17*	−0.09	−0.25*	−0.40*	0.15*	(0.89)		
(10) NC-MTL	5.05	1.15	0.27*	0.02	0.36*	−0.16*	0.18*	0.28*	0.49*	−0.14*	−0.46*	(0.89)	
(11) PS-MTL	6.12	0.77	0.40*	0.09	0.24*	−0.18*	0.23*	0.22*	0.47*	−0.04	−0.29*	0.49*	(0.89)

*N* = 248. Cronbach’s Alpha is on the diagonal in the parentheses for psychometric variables. Gender was coded as 1 = female, 0 = male; Academic degree (1 = yes, 2 = no); First-gen = first-generation college student (1 = yes, 0 = no); PLE = Past leadership experience (1 = yes, 0 = no); SSRM = same-sex role model (1 = yes, 0 = no); FLSA = female leadership strength awareness; AFF-MTL = affective-identity motivation to lead; SN-MTL = social normative motivation to lead; NC-MTL = non-calculative motivation to lead; PS-MTL = prosocial motivation to lead. *p < 0.05.

We further applied our 6-item scale developed in Study 1 measuring individual differences in PS-MTL (alpha = 0.89; omega = 0.93; CR = 0.90; AVE = 0.57). A confirmatory factor analysis verified the one-dimensional structure of PS-MTL for most of the indicators (CFI = 0.96; TLI = 0.94; RMSEA = 0.10; SRMR = 0.04).

Participants’ *gender* was assessed using a four-category item with the response options: ‘female,’ ‘male,’ ‘diverse,’ and ‘prefer not to say’. As no participants selected ‘diverse’ or ‘prefer not to say’, gender was dichotomized for analysis, with ‘female’ coded as 1 and ‘male’ coded as 0.

##### Same-sex role models (SSRM)

Following Elprana et al. (2015), we asked our participants whether their parents held a leadership position. We created a dummy variable for same-sex role model availability (SSRM), coded as 1 if participants—regardless of gender—reported having at least one same-sex parent in a leadership role, and 0 if no same-sex role model was available. The variable did not distinguish between participants with one or both parents as same-sex role models, nor between male and female participants. In our sample, 42 males and 114 females had a same-sex role model; 37 males and 55 females did not have a same-sex role model.

##### Control variables

We controlled for participants’ *age*, since older students may have gained more leadership experience than younger (e.g., during their career in school, university or sports) and thus age may be positively related to MTL (Chan and Drasgow, 2001). Since education is a central predictor for a leadership career (Hüttges and Fay, 2015), we assume that participants with a higher academic degree (e.g., students already holding a BA’s degree) are more likely to strive for a leadership career and thus show higher levels of MTL than participants with lower academic degrees. Thus, *academic degree* (i.e., “Do you already have a university or college degree?”) was included as control variable. Given that parents holding an academic degree are more likely to be perceived as successful leadership role models than parents without an academic degree (Neumeyer and Alesi, 2018), we included *first-generation college student* (i.e., “Both of my parents do not have an academic degree”) as control variable. In addition, we controlled for *past leadership experience* which has been found as predictive of MTL in previous studies (e.g., Chan and Drasgow, 2001; Badura et al., 2020).

#### Sample and preliminary analyses

Our independent sample included *N* = 248 students, of which 169 identified with the female gender, and 79 with the male gender. No participant reported a diverse gender identity or chose to withhold a response. Mean age was 23.27 years (SD = 3.86) with 2.6 completed semesters on average (SD = 1.37), and most of the participants were not first-generation college students (*m* = 0.28; SD = 0.45; measured as dummy variable with 1 = Yes; 0 = No). Notably, most of the participants had already gained some leadership experience (*m* = 0.71; SD = 0.45; coded by a dummy variable with 1 = Yes; 0 = No).

Table 5 offers descriptive statistics for all study variables. As expected, the MTL types with primarily agentic orientation (i.e., AFF-MTL and SN-MTL) were significantly positively correlated (*r* = 0.15; *p* < 0.05); the same is true for the MTL types with primarily communal orientation (i.e., NC-MTL and PS-MTL; *r* = 0.49; *p* < 0.05). Gender was correlated with the MTL types in that female gender was positively related to both NC-MTL (*r* = 0.27; *p* < 0.05) and PS-MTL (*r* = 0.40; *p* < 0.05) and negatively to both AFF-MTL (*r* = −0.16; *p* < 0.05) and SN-MTL (*r* = −0.31; *p* < 0.05). FLSA and SSRM were only slightly inter-correlated (*r* = 0.25; *p* < 0.05), confirming the distinctness of our two moderators.

Before testing our hypotheses, we conducted confirmatory factors analyses to assure the distinctiveness of the four MTL constructs used in this study. As Table 6 shows, the 4-factor model, distinguishing AFF-MTL, SN-MTL, NC-MTL, and PS-MTL, reached better fit indices (*X*^2^ = 740.50, df = 293; CFI = 0.92; TLI = 0.91; RMSEA = 0.08; SRMR = 0.07) than the alternative models. In the CFA, PS-MTL items were consistently loaded onto the AFF-MTL factor in all models except the 4-factor model, where each MTL dimension was modeled as a distinct latent construct. Similarly, SN-MTL items were combined with AFF-MTL in the 1-factor-model and the 2-factor model, whereas NC-MTL was treated as a separate factor in both the 2-factor-model and the 3-factor model. Only in the 4-factor model were AFF-MTL, NC-MTL, SN-MTL, and PS-MTL each represented as independent latent constructs. In particular, the 3-factor model, which suggests that the items of the newly developed scale for PS-MTL would just be completely absorbed by the original AFF-MTL scale by Chan and Drasgow (2001), reached significantly worse fit indices (*X*^2^ = 1627.85, df = 296; CFI = 0.74; TLI = 0.71; RMSEA = 0.14; SRMR = 0.17) than the 4-factor model.

**Table 6:** Results of confirmatory factor analysis of study variables.

Model	*X*^2^ (df)	Δ*X*^2^	CFI	TLI	RMSEA	SRMR	Loading range	Inter-factor correlation
1-factor	3008.499*** (299)		0.46	0.42	0.19	0.24	0.04–1.05	–
2-factor	2356.792*** (298)	651.707	0.59	0.55	0.17	0.22	0.03–1.04	−0.12
3-factor	1627.850*** (296)	728.942	0.74	0.71	0.14	0.17	0.02–1.03	−0.70 – 0.39
4-factor	740.50*** (293)	887.350	0.92	0.91	0.08	0.07	0.63–1.03	−0.70 – 0. 44

*N* = 248; Estimation method ml; df = degrees of freedom; *X*^2^ = chi-square; Δ*X*^2^ = difference in chi-square; CFI = Comparative Fit Index; TLI = Tucker–Lewis Index; SRMR = Standardized Root Mean Square Residual; RMSEA = Root Mean Square Error of Approximation; Model 1 (1-factor model) combines all four variables as one factor. Model 2 (2-factor model) includes AFF-MTL and NC-MTL, loading items of SN-MTL and PS-MTL onto AFF-MTL. Model 3 (3-factor model) includes AFF-MTL, NC-MTL, and SN-MTL, loading items of PS-MTL onto AFF-MTL. Model 4 (4-factor model) includes all four variables being treated as independent factors. All *X*^2^ (df)s are significant at ***p < 0.001 level.

## Results

Since our data was non-normally distributed as evidenced by a significant Shapiro–Wilk-test, we used a Mann–Whitney *U*-Test to examine our first hypothesis accordingly (Orcan, 2020). As shown in Table 7, the test provided partial support for Hypothesis 1. While male participants showed both more AFF-MTL (although not significantly for the *U*-Test; see Hypothesis 1a) and more SN-MTL (see Hypothesis 1b) than female participants, the latter showed significantly higher levels of both NC-MTL (see Hypothesis 1c) and PS-MTL (see Hypothesis 1d) for females than their male colleagues. These findings provide evidence of gender-related differences in MTL, supporting Hypothesis 1b–d. By trend, our results thus correspond to the findings of the meta-analysis by Badura et al. (2020). In other words, women tend to have different motives for taking on leadership roles than men.

**Table 7:** Mann–Whitney *U*-test: summary of differences between male and female.

Variables	Males		Females		*U*	*Z*
	Mean rank	Median	Mean rank	Median		
AFF-MTL	134.9	4.78	119.64	4.56	5854.00	1.562
SN-MTL	152.74	4.25	111.3	3.875	4444.50	4.243***
NC-MTL	95.0	4.71	138.31	5.43	4341.50	−4.441***
PS-MTL	89.14	5.83	141.03	6.33	3,882	−5.334***
FLSA	74.42	4.25	147.91	5.75	2719.5	−7.535***

*N* = 248; AFF-MTL = affective-identity motivation to lead; SN-MTL = social normative motivation to lead; NC-MTL = non-calculative motivation to lead; PS-MTL = prosocial motivation to lead. ***p < 0.001.

We tested Hypothesis 2 and Hypothesis 3 by using hierarchical linear regression analyses. To address violations of normality and heteroscedasticity in our regression residuals, we employed robust standard errors consistent with recent recommendations (Baissa and Rainey, 2020; Thinh et al., 2020). The hypotheses were tested by regressing the four MTL types (i.e., AFF-MTL, SN-MTL, NC-MTL, and PS-MTL) on the controls (i.e., age, first-generation student, academic degree, past leadership experience), the main effects (i.e., gender, SSRM, FLSA), and the respective interaction terms (i.e., gender and SSRM; gender and FLSA). For AFF-MTL, no significant interaction effect was shown (see Table 8, Model 2), failing to confirm Hypothesis 2a and Hypothesis 3a. In contrast, Table 8 shows significant interaction effects for SN-MTL with SSRM (ß = −0.67, *p* < 0.01; see Model 4) and with FLSA (ß = −0.44, *p* < 0.001; see Model 4), for NC-MTL with SSRM (ß = 0.64, p < 0.05; see Model 6) and with FLSA (ß = 0.75, *p* < 0.001; see Model 6), and for PS-MTL with SSRM (ß = 0.57, *p* < 0.05; see Model 8) and with FLSA (ß = 0.28, *p* < 0.01; see Model 8).

**Table 8:** Results of regression analyses for motivation to lead factors.

Variables	(1)	(2)	(3)	(4)	(5)	(6)	(7)	(8)
	AFF-MTL	AFF-MTL	SN-MTL	SN-MTL	NC-MTL	NC-MTL	PS-MTL	PS-MTL
Intercept	−0.51	−0.61	0.46	0.94*	0.20*	−0.37	−0.74*	−0.88**
	(0.58)	(0.61)	(0.30)	(0.36)	(0.55)	(0.47)	(0.35)	(0.29)
Gender	−0.33	−0.16	−0.65***	−0.27	0.40**	0.14	0.58***	0.23
	(0.17)	(0.24)	(0.14)	(0.18)	(0.16)	(0.19)	(0.12)	(0.16)
Age	0.05*	0.05	0.01	−0.02	−0.04	−0.02	0.00	0.02
	(0.03)	(0.03)	(0.01)	(0.01)	(0.02)	(0.02)	(0.02)	(0.01)
Academic degree	−0.70**	−0.71**	−0.42*	−0.11	0.85***	0.47**	0.21*	0.00
	(0.25)	(0.26)	(0.18)	(0.19)	(0.16)	(0.15)	(0.10)	(0.10)
First-gen	0.03	0.04	0.31*	−0.08	−0.21	0.21	−0.17	0.02
	(0.19)	(0.21)	(0.14)	(0.15)	(0.15)	(0.17)	(0.11)	(0.12)
PLE	−0.22	−0.23	−0.12	0.05	0.32*	0.12	0.30**	0.21*
	(0.19)	(0.20)	(0.16)	(0.16)	(0.15)	(0.13)	(0.10)	(0.10)
SSRM		0.15		0.10		−0.20		−0.28
		(0.27)		(0.16)		(0.26)		(0.19)
Gender X SSRM		−0.32		−0.67**		0.64*		0.57*
		(0.34)		(0.23)		(0.30)		(0.24)
FLSA		−0.01		0.07		−0.21		−0.02
		(0.14)		(0.09)		(0.12)		(0.08)
Gender X FLSA		0.10		−0.44***		0.75***		0.28**
		(0.17)		(0.12)		(0.14)		(0.09)
*R* ^2^	0.07	0.07	0.14	0.25	0.21	0.43	0.22	0.35
Δ*R*^2^		0.00		0.11		0.22		0.13
*R*^2^ Adjusted	0.05	0.03	0.11	0.22	0.19	0.41	0.20	0.32
*F*	3.01*	2.12*	9.20***	7.08***	15.83***	27.08***	14.23***	20.24***

*N* = 248; robust standard errors in parentheses; The baseline for ΔR2 is established by Model 1, Model 3, Model 5, and Model 7, respectively. Gender was coded as 1 = female, 0 = male; Academic degree (1 = yes, 0 = no); First-gen = first-generation college student (1 = yes, 0 = no); PLE = past leadership experience (1 = yes, 0 = no); SSRM = same-sex role model (1 = yes, 0 = no); FLSA = female leadership strength awareness; AFF-MTL = affective-identity motivation to lead; SN-MTL = social normative motivation to lead; NC-MTL = non-calculative motivation to lead; PS-MTL = prosocial motivation to lead. *p < 0.05, **p < 0.01, ***p < 0.001.

**Figure 1:**
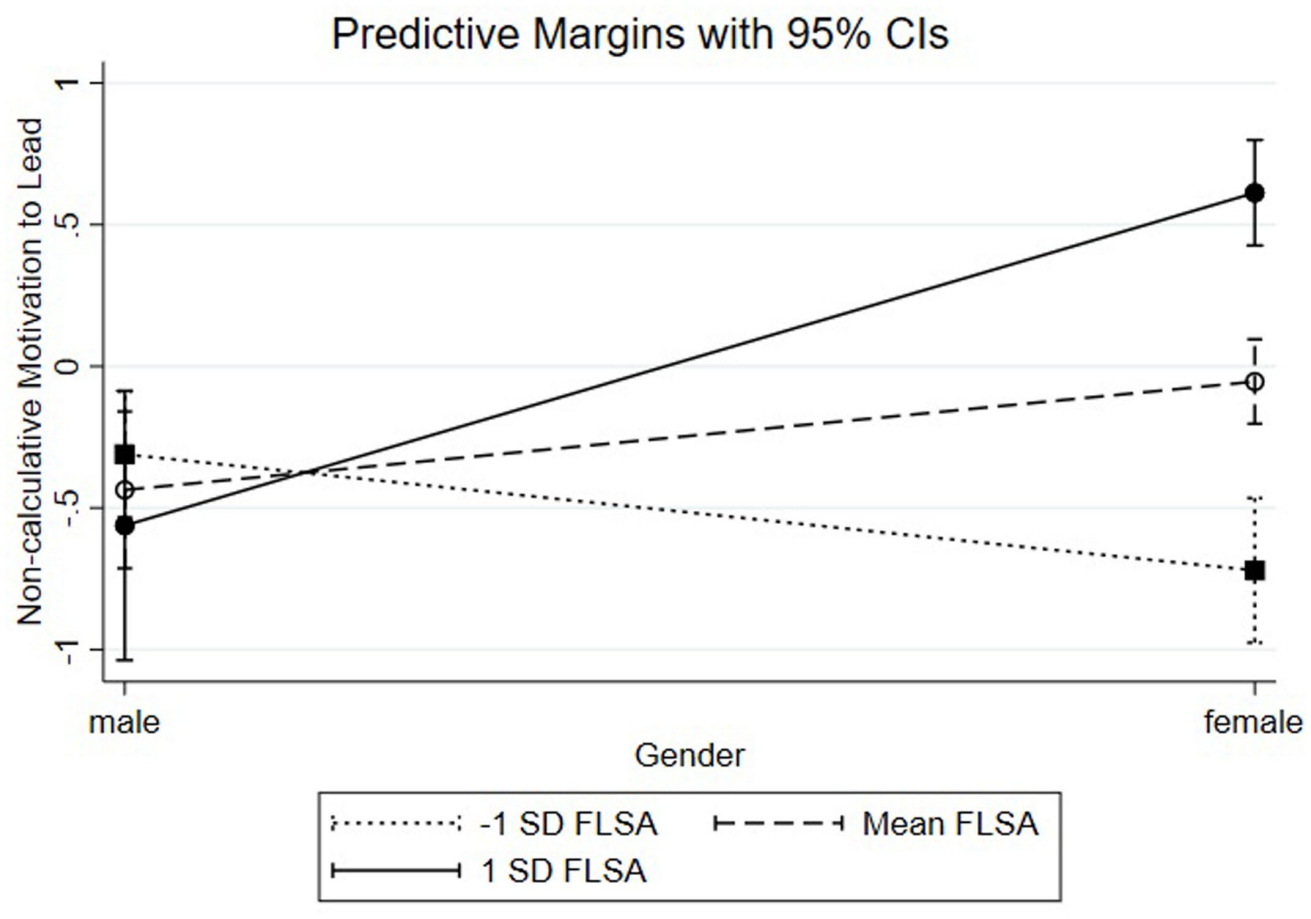
Non-calculative motivation to lead by gender and FLSA.

To analyze the shape of the significant interaction effects, we conducted simple slope calculations (Aiken and West, 1991; Dawson, 2014). Considering SN-MTL (see Figure 1), the slope analysis for women with SSRM revealed a significantly negative SN-MTL relationship (ß = −0.51, SE = 0.12, *p* < 0.001) while women without SSRM showed a significantly positive SN-MTL relationship (ß = 0.33, SE = 0.17, *p* < 0.05). Hypothesis 2b which predicted that women with SSRM will have higher levels of SN-MTL than women without SSRM is therefore not confirmed.

**Figure 2:**
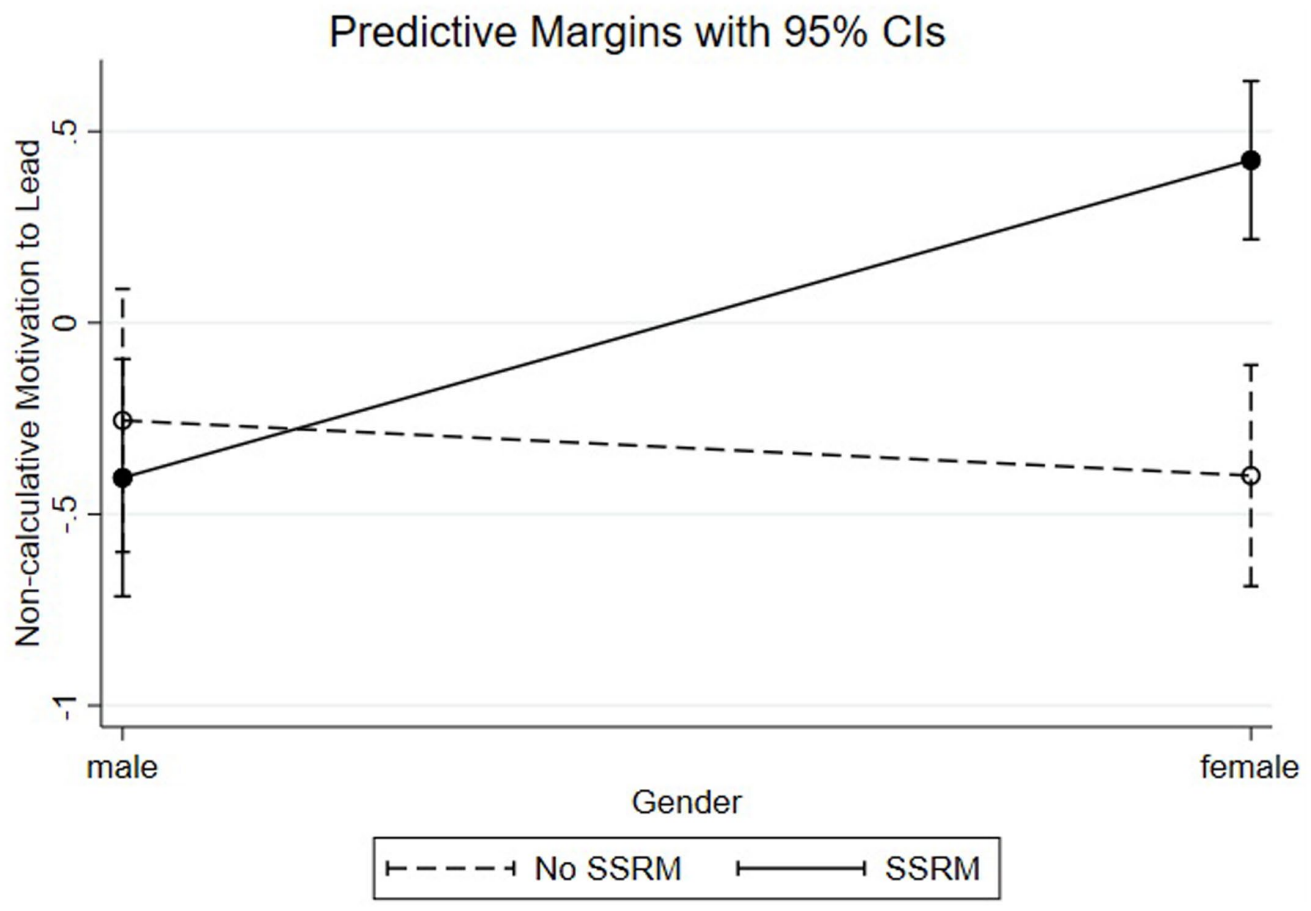
Non-calculative motivation to lead by gender and SSRM.

For NC-MTL (see Figure 2), we computed the slope for women with SSRM (ß = 0.42, SE = 0.10, *p* < 0.001) and women without SSRM (ß = −0.40, SE = 0.15, *p* < 0.01). These results indicate that female participants with a SSRM available have higher levels of NC-MTL than female participants without a SSRM, supporting Hypothesis 2c.

**Figure 3:**
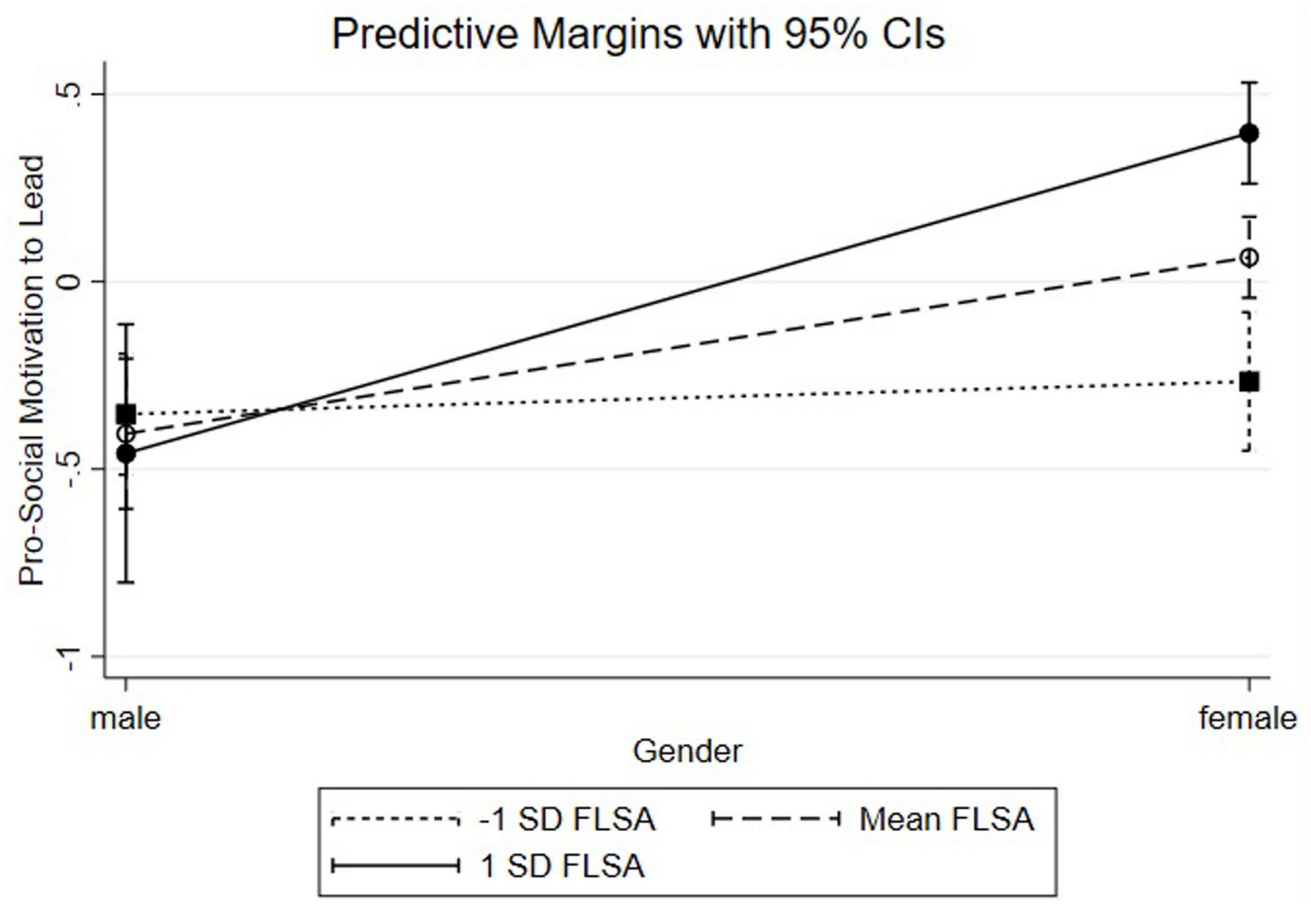
Pro-social motivation to lead by gender and FLSA.

For PS-MTL, we investigated a simple slope computation (see Figure 3) for women with SSRM (ß = 0.35, SE = 0.07, *p* < 0.001) and women without SSRM (ß = −0.12, SE = 0.10, ns). These results indicate that female participants with SSRM have higher levels of PS-MTL than those without SSRM, supporting Hypothesis 2d.

**Figure 4:**
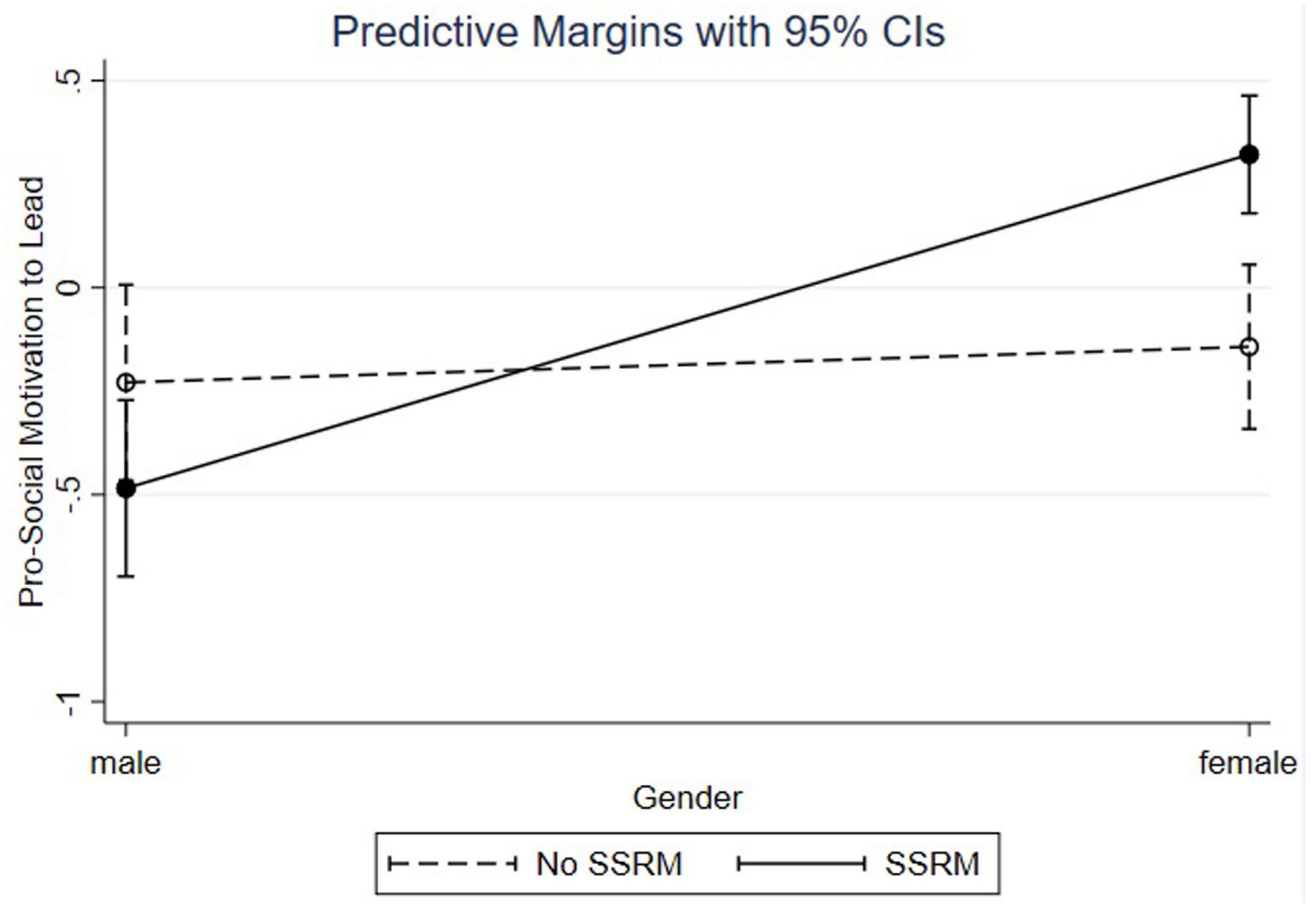
Pro-social motivation to lead by gender and SSRM.

Concerning Figure 4, the simple slope analysis shows that women with high levels of FLSA (1 SD above the mean; ß = −0.57, SE = 0.11, *p* < 0.001) have lower levels of SN-MTL than women with moderate levels of FLSA (at the mean; ß = −0.07, SE = 0.09, ns) or lower levels of FLSA (1 SD below the mean; ß = 0.43, SE = 0.16, *p* < 0.01), rejecting Hypothesis 3b.

**Figure 5:**
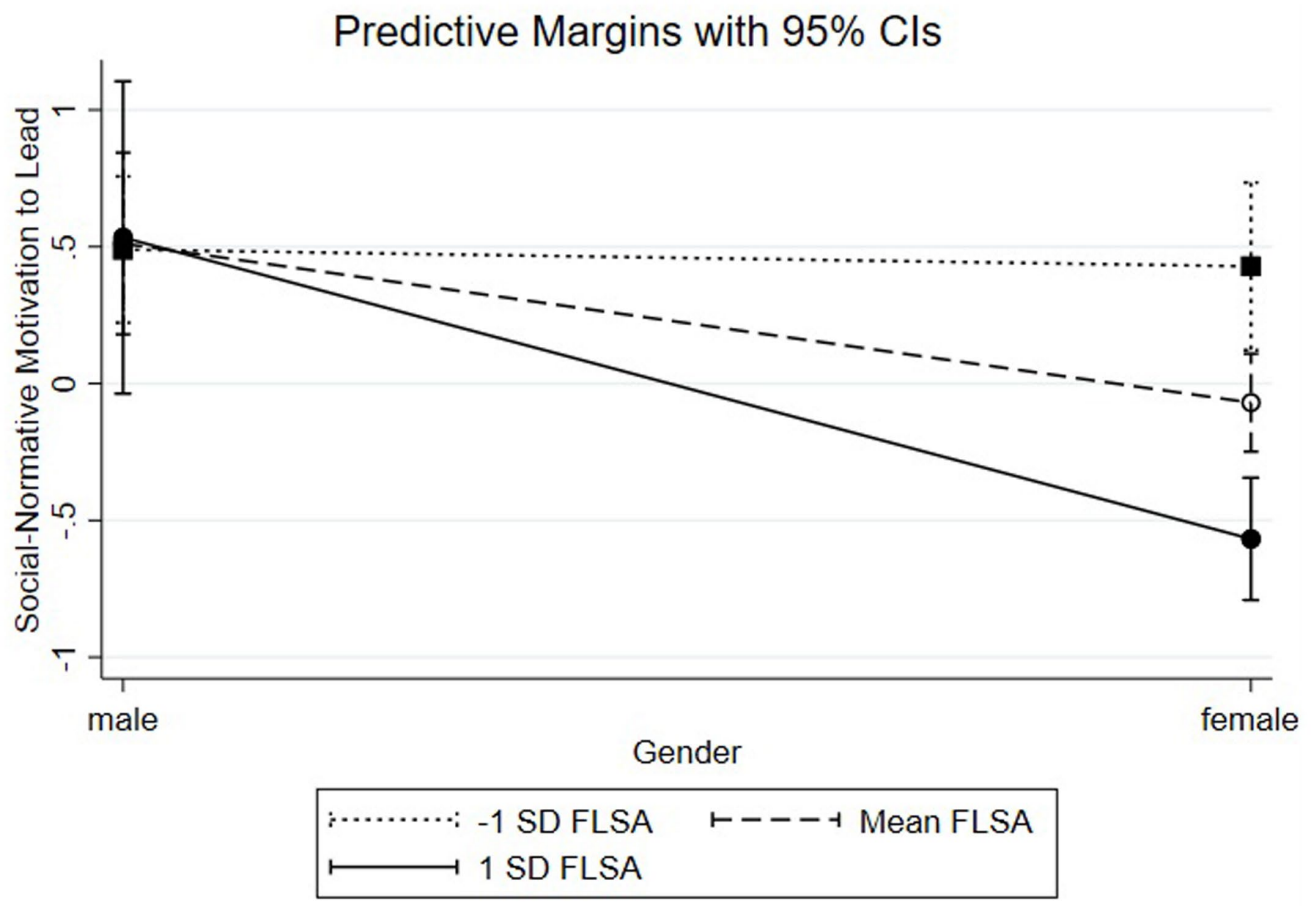
Social-normative motivation to lead by gender and FLSA.

As demonstrated in Figure 5, a simple slope analysis revealed that women with high levels of FLSA (1 SD above the mean; ß = 0.67, SE = 0.09, *p* < 0.001) have higher levels of NC-MTL than women with moderate levels of FLSA (at the mean; ß = −0.07, SE = 0.07, ns) or lower levels of FLSA (1 SD below the mean; ß = −0.80, SE = 0.12, *p* < 0.001), supporting Hypothesis 3c.

**Figure 6:**
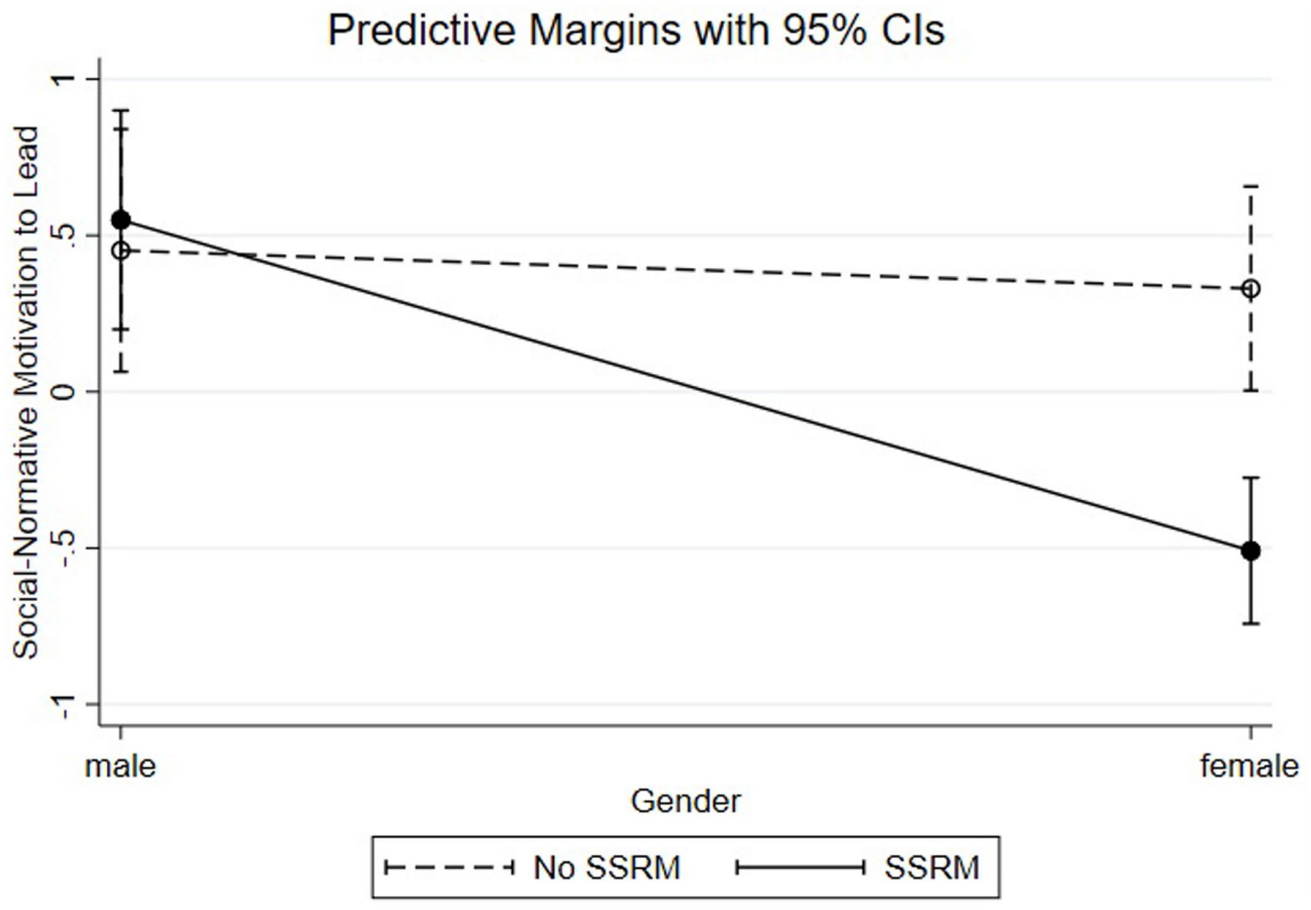
Social-normative motivation to lead by gender and SSRM.

The simple slope analysis shown in Figure 6 reveals that women with high levels of FLSA (1 SD above the mean; ß = 0.45, SE = 0.07, *p* < 0.001) have higher levels of PS-MTL than women with moderate (at the mean, ß = 0.09, SE = 0.05, ns) or lower levels of FLSA (1 SD below the mean; ß = −0.28, SE = 0.09, *p* < 0.001), supporting Hypothesis 3d.

## Discussion

Motivation to lead has been confirmed to predict both leadership emergence and leadership effectiveness (Badura et al., 2020). The female leadership gap (Powell and Graves, 2018) may thus partially result from a female disadvantage in MTL. In fact, the most recent meta-analysis reports a small female disadvantage in AFF-MTL and SN-MTL and a small female advantage in NC-MTL (Badura et al., 2020). In this paper, we argue that Chan and Drasgow’s (2001) conception of MTL may be incomplete and biased in that women’s motivation to take on leadership roles is underrepresented. The female disadvantage in MTL reported in the literature may thus be an artifact of conceptualizing and measuring MTL.

To overcome this possible gender bias in understanding MTL, we propose PS-MTL, that is, making a positive difference in other people’s lives by taking a leadership role, as a fourth MTL type and provide a preliminary validation of this variable (see Study 1). In line with Badura et al. (2020), Study 2 reveals that women report slightly lower levels of affective–identity motivation to lead (AFF-MTL) and significantly lower levels of social–normative motivation to lead (SN-MTL), while exhibiting significantly higher levels of non–calculative motivation to lead (NC-MTL) compared to men. In addition, women show significantly higher degrees of PS-MTL than men (supporting Hypothesis 1b–d). More precisely, compared to the other MTL types, the gender difference in MTL is highest in PS-MTL. The conventional three-dimensional MTL construct (Chan and Drasgow, 2001) may thus have underestimated female MTL. The first contribution of our study is therefore to introduce a conceptualization of MTL that equally incorporates both male and female strengths in MTL. In contrast to recent work on leadership aspiration (Netchaeva et al., 2022), our results suggest that women are not less motivated to take on leadership positions, but they seem to be differently motivated than men.

Indeed, leadership is inherently a multidimensional construct, and leadership motivation theories should capture the full range of motivational drivers—particularly those grounded in social, relational, and altruistic goals. The concept of PS-MTL is firmly situated within established traditions in leadership theory that emphasize leadership as a relational, value-driven process (e.g., transformational, servant, and ethical leadership; Northouse, 2016). Furthermore, our multi-sample validation efforts provide robust evidence that PS-MTL is both psychometrically distinct from existing MTL components and predictively valuable.

In this context, gender functions not as a normative objective but as a diagnostic lens: it reveals where current theoretical models may inadvertently privilege certain motivational frameworks—typically agentic, individualistic, and self-referential—over others that are communally oriented or prosocial in nature. Foundational theoretical work has long shown that agentic and communal motives are both core to human motivation, but only agentic dimensions—such as assertiveness, dominance, and independence—have historically been emphasized in leadership models (Locke, 2018; London et al., 2019). This overrepresentation of agentic traits aligns with masculine-coded leadership stereotypes, often to the exclusion of communal orientations (Vial and Cimpian, 2024). Moreover, followers themselves increasingly value communal traits such as empathy and collaboration in their leaders—traits traditionally underemphasized in theory (Ponce de Leon and Bailey, 2025). When substantial empirical evidence suggests that a particular population segment (e.g., women) consistently expresses motivational patterns that are not adequately captured by existing constructs, this points to a theoretical blind spot, not merely a gap in scale design.

Secondly, we investigate ways to further strengthen female MTL. We demonstrate that women who perceive same-sex role models (SSRM) have higher levels of both NC-MTL and PS-MTL (Hypothesis 2c and 2d). Fritz and van Knippenberg (2020) were able to identify role modeling as a relevant factor for female leadership aspiration; Elprana et al. (2015) analyzed same-sex role models as a mediator of the relationship between gender and AFF-MTL. Against this backdrop, our study is the first to investigate same-sex role models as a moderator in the relationship between gender and all four MTL types (i.e., AFF-MTL, SN-MTL, NC-MTL, and PS-MTL). In addition, we introduce female leadership strength awareness (FLSA) as a novel variable and provide a preliminary validation of this variable (see Study 1). Women who are aware of the general strength of female leaders develop higher levels of both NC-MTL and PS-MTL (Hypothesis 3c and 3d). FLSA even works if no SSRM is available that meets the criteria of (1) similarity, (2) relevance and (3) attainability (Sealy and Singh, 2009).

Our second contribution is thus to introduce both SSRM and FLSA as moderators of the relationship between gender and MTL. While previous studies mainly focused on the incongruity between male leader stereotype and female gender stereotype and were striving to compensate female disadvantages and weakness (e.g., Eagly and Carli, 2003), our study points at particular strengths of female leaders. We thus systematically introduce the notion of a female leadership advantage into the literature on female MTL.

Interestingly, the moderation effects of both SSRM and FLSA could only be shown for NC-MTL and PS-MTL (Hypotheses 2c and 3c and 2d and 3d)—the MTL types which are more pronounced in women than in men. In contrast, for AFF-MTL and SN-MTL, for which women have lower levels than men, no such moderation effect was found. Accordingly, Hypotheses 2a and 2b as well as Hypotheses 3a and 3b could not be supported. This finding may be interpreted as another clue that female MTL may be different from male MTL. To stimulate female AFF-MTL and SN-MTL, other variables (e.g., work-life-initiatives; Fritz and van Knippenberg, 2017) could be investigated as moderators. In other words, our results speak to the idea that measures taken to promote female MTL will particularly stimulate those motives which seem to be more “typical” of female than male leaders.

### Limitations and implications for further research

First, we tested our hypotheses using a convenience sample of 248 students at German universities. Although our results resonate with the recent meta-analysis on MTL (Badura et al., 2020) and former studies on the favorable role of SSRM for MTL (Elprana et al., 2015; Fritz and van Knippenberg, 2020), they cannot be generalized. In particular, it is unclear if our results are valid for samples from different countries and cultures. According to gender role theory, the chances for women to overcome the “leadership labyrinth” (Eagly and Carli, 2007) are strongly related to the level of gender stereotypes and the working conditions for women (Devnew et al., 2018), hence to levels of gender egalitarity in the respective society. However, levels of gender egalitarity continue to differ largely between countries (World Economic Forum, 2024). In order to test if female MTL generally differs from male motivation as demonstrated in this study, further research in other countries would be thus needed.

In addition, it is questionable if the results of our study on students are valid for employees as well. However, student samples and samples of working adults have almost equally been used to investigate MTL (Badura et al., 2020). Moreover, the authors did only find few moderating effects of sample type (i.e., students vs. working adults), limited to some antecedent factors of MTL. The reason for this finding may be that many students already have some degree of work and leadership experience (as was the case in our sample).

Second, given the cross-sectional design of our study, our analyses do not yield causal results. The moderated relationships between gender and MTL as hypothesized (see Hypothesis 2 and Hypothesis 3) may be modeled in reversed order. However, for logical arguments, reversed orders are not likely. The assessment of the gender variable in our study is not likely to be dependent on participants’ level of MTL. On the contrary, there are reasons to assume that women have lower levels of MTL (Badura et al., 2020). In addition, our cross-sectional approach is in line with previous studies on gender differences in MTL and leadership aspiration, respectively (Badura et al., 2020; Elprana et al., 2015; Fritz and van Knippenberg, 2017, 2020; Porter et al., 2016). Nevertheless, experimental or longitudinal designs are needed to test the assumed causal relationship and to rule out effects of other variables.

Third, we introduced PS-MTL and FLSA as newly developed constructs (see Study 1). PS-MTL as a fourth type of MTL was developed according to Chan and Drasgow’s (2001) three-dimensional MTL construct. More precisely, analogously to the existing scales for AFF-MTL, SN-MTL, and NC-MTL, we provided a 6-item scale representing potential prosocial motives for taking on a leadership role. FLSA was designed according to empirical findings on the female leadership advantage (Boerner, 2023). In order to transform these findings into aspects of the participant’s awareness of the female leadership advantage, they were transformed into items of a 4-item scale. Although the results of our preliminary validation studies for both PS and FLSA with independent samples are promising, the newly developed scales for these constructs need to be tested in further studies.

### Practical implications

If the results of our study could be repeated in further research, a female disadvantage in MTL can be excluded as a significant reason for the female leadership gap (Powell and Graves, 2018). Rather, our findings suggest that women are not less motivated to lead than men, but their motives for taking on leadership roles differ from those of their male colleagues. Since the more social motives represented in PS-MTL (i.e., to benefit others by taking a leadership role) may prevent leaders from using unethical and abusive leadership behaviors, this result may even speak to a female advantage in MTL.

In order to use this advantage for reducing the female leadership gap, one measure would be to underline the prosocial aspects of the leadership role. Apparently, women are less motivated to take on leadership roles in order to exert power over followers (Shen and Joseph, 2021) or to achieve individual benefit (i.e., they have higher levels of NC-MTL than men); instead, they are likely to take on leadership roles despite their individual costs and for prosocial reasons. In order to stimulate women’s MTL, the prosocial aspects of a leadership role could be emphasized more strongly. For example, job advertisements could elaborate more on the societal role of the recruiting company. In addition, the prosocial aspects of leadership such as supporting and developing followers could be promoted more strongly, in order to “shift away from a traditional masculine view of leadership and toward a more feminine […] outlook” (Paustian-Underdahl et al., 2014, p. 1131).

In addition, our results show that female motivation to lead can be further enhanced by gender-sensitive approaches (Gierke et al., 2025) such as providing SSRM and by popularizing the notion of a FLSA. In other words, by emphasizing that many women are successful in leadership positions and that women have general strengths in leadership, more female employees could be encouraged to take on leadership roles. As the meta-analysis by Shen and Joseph (2021) reveals, women are seen as more effective leaders in other’s rankings (i.e., by managers, employees or neutral observers) – while they are less effective leaders in self-rankings (i.e., female leaders see themselves as less effective than male leaders evaluate themselves; Paustian-Underdahl et al., 2014). Similarly, women tend to have lower levels of leadership self-efficacy than men (Dwyer, 2019) and higher levels of the so-called imposter syndrome (Langford and Clance, 1993; Tewfik et al., 2024). Thus, informing and advising both male and female managers and employees about female strengths in leadership seems to be a central measure to enhance female MTL. Thereby, potential female candidates could strengthen their leadership self-efficacy, which is one of the strongest predictors of both MTL and leadership effectiveness (Badura et al., 2020; Chan and Drasgow, 2001).

Admittedly, the suggested measures to overcome the female leadership gap discussed so far mainly address women’s self-assessments as (possible) leaders. It should therefore be noted that gender stereotypes, although declining (Koenig et al., 2011), are still one of the most serious barriers to female careers in leadership positions (Heilman et al., 2024), as well as other factors such as devaluating of women, organizational culture and processes, work-family-conflict and the leaky pipeline (van’t Foort-Diepeveen et al., 2021). Thus, measures to reduce the female leadership gap should not be limited to initiatives empowering potential female candidates themselves, but need to be addressed at the organizational and societal levels as well (Gierke et al., 2025; Metz and Kumra, 2019; Rybnikova and Menzel, 2021).

## Data Availability

The raw data supporting the conclusions of this article will be made available by the authors, without undue reservation.
