# Supplementary material for: Female motivation to lead: the impact of same-sex role models and female leadership strength awareness
Source: Front Psychol. 2025 Aug 7;16:1544411. doi: 10.3389/fpsyg.2025.1544411 (PMC12372470; doi:10.3389/fpsyg.2025.1544411)
Supplement: Supplementary file 1 [file Table_1.docx]

**Appendix A. Prosocial Motivation to Lead (PS-MTL)**

**Table 1**

*Descriptive Statistics and Correlations of Study Variables (Sample 2)*

| Variables | Mean | SD | (1) | (2) | (3) | (4) | (5) | (6) | (7) | (8) | (9) | (10) | |
| --- | --- | --- | --- | --- | --- | --- | --- | --- | --- | --- | --- | --- | --- |
| (1) Gender | 0.64 | 0.48 | - |  |  |  |  |  |  |  |  |  |  |
| (2) Age | 30.4 | 13.1 | 0.04 | - |  |  |  |  |  |  |  |  |  |
| (3) Academic degree | 0.40 | 0.49 | 0.00 | 0.47* | - |  |  |  |  |  |  |  |  |
| (4) PLE | 0.69 | 0.46 | 0.03 | 0.25* | 0.11 | - |  |  |  |  |  |  |  |
| (5) Leadership Self-Efficacy | 4.37 | 0.44 | 0.03 | -0.04 | -0.12 | 0.09 | (0.91) |  |  |  |  |  |  |
| (6) Leadership Aspiration | 4.95 | 0.83 | 0.09 | -0.08 | -0.08 | 0.22* | 0.35* | (0.82) |  |  |  |  |  |
| (7) AFF-MTL | 4.76 | 1.04 | 0.03 | 0.07 | -0.04 | 0.33* | 0.28* | 0.43* | (0.89) |  |  |  |  |
| (8) SN-MTL | 4.40 | 0.91 | 0.00 | -0.07 | -0.03 | 0.20* | 0.28* | 0.35* | 0.33* | (0.73) |  |  |  |
| (9) NC-MTL | 4.45 | 0.83 | 0.12 | 0.26* | 0.13 | -0.04 | 0.01 | -0.05 | 0.02 | -0.10 | (0.85) | |  |
| (10) PS-MTL | 5.93 | 0.72 | 0.14* | -0.15* | -0.06 | 0.17* | 0.29* | 0.40* | 0.29* | 0.37* | 0.13 | | (0.85) |
| Note. *N* = 200-212 (Academic degree had only 200 observations). Cronbach’s alpha is on the diagonal in the parentheses for psychometric variables. Gender was coded as 1 = female, 0 = male; Academic degree (1 = yes, 0 = no); PLE = past leadership experience (1 = yes, 0 = no); AFF-MTL = affective-identity motivation to lead; SN-MTL = social normative motivation to lead; NC-MTL = noncalculative motivation to lead; PS-MTL = prosocial motivation to lead. *p < .05 | | | | | | | | | | | | | |

**Table 2**

*Average-Variance-Extracted, Square-Root Correlations, and HTMT (Sample 2)*

| Variables | (1) | (2) | (3) | (4) | (5) | (6) |
| --- | --- | --- | --- | --- | --- | --- |
| (1) Leadership Self-Efficacy | *0.69* | 0.72 | 0.77 | 0.49 | 0.11 | 0.33 |
| (2) Leadership Aspiration | 0.51 | *0.59* | 0.64 | 0.54 | 0.04 | 0.36 |
| (4) AFF-MTL | 0.59 | 0.41 | *0.49* | 0.48 | 0.05 | 0.22 |
| (5) SN-MTL | 0.24 | 0.29 | 0.23 | *0.36* | 0.05 | 0.49 |
| (6) NC-MTL | 0.01 | 0.0 | 0.0 | 0.0 | *0.59* | 0.27 |
| (7) PS-MTL | 0.11 | 0.13 | 0.05 | 0.24 | 0.07 | *0.51* |
| *N = 212*; Coefficients below the diagonal indicate squared correlations. Coefficients in Italic at the diagonal indicate the AVE. Coefficients above the diagonal indicate the HTMT ratio. AFF-MTL = affective-identity motivation to lead; SN-MTL = social normative motivation to lead; NC-MTL = noncalculative motivation to lead; PS-MTL = prosocial motivation to lead. | | | | | | |

**Table 3**

*Descriptive Statistics and Correlations of Study Variables (Sample 3)*

| Variables | Mean | SD | (1) | (2) | (3) | (4) | (5) | (6) | (7) | (8) | (9) | (10) | (11) | (12) |  |
| --- | --- | --- | --- | --- | --- | --- | --- | --- | --- | --- | --- | --- | --- | --- | --- |
| (1) Gender | 1.44 | 0.50 | - |  |  |  |  |  |  |  |  |  |  |  |  |
| (2) Age | 23.4 | 4.13 | 0.07 | - |  |  |  |  |  |  |  |  |  |  |  |
| (3) PLE | 0.56 | 0.50 | 0.01 | 0.10 | - |  |  |  |  |  |  |  |  |  |  |
| (4) Academic Degree | 0.23 | 0.42 | 0.03 | 0.44* | 0.11 | - |  |  |  |  |  |  |  |  |  |
| (5) First-gen | 0.47 | 0.50 | 0.02 | 0.13 | -0.09 | 0.08 | - |  |  |  |  |  |  |  |  |
| (6) Leadership Self-Efficacy | 4.40 | 0.53 | 0.06 | -0.02 | 0.28* | 0.14* | -0.11 | - |  |  |  |  |  |  |  |
| (7) Leadership Aspiration | 4.91 | 0.85 | 0.10 | -0.04 | 0.21* | 0.03 | -0.04 | 0.38* | - |  |  |  |  |  |  |
| (8) AFF-MTL | 4.78 | 1.14 | 0.04 | -0.01 | 0.30* | 0.04 | -0.03 | 0.34* | 0.55* | (0.88) |  |  |  |  |  |
| (9) SN-MTL | 4.48 | 1.06 | 0.03 | -0.05 | 0.14* | 0.05 | -0.07 | 0.36* | 0.39* | 0.34* | (0.74) |  |  |  |  |
| (10) NC-MTL | 3.39 | 1.14 | -0.00 | 0.02 | -0.00 | 0.03 | -0.01 | -0.02 | 0.05 | -0.05 | -0.07 | (0.83) |  |  |  |
| (11) PS-MTL | 5.94 | 0.76 | -0.07 | -0.03 | 0.08 | -0.02 | 0.03 | 0.26* | 0.29* | 0.30* | 0.38* | -0.35* | (0.86) |  |  |
| (12) Leader effectiveness | 4.62 | 1.03 | -0.03 | 0.05 | 0.24* | 0.07 | -0.01 | 0.46* | 0.48* | 0.59* | 0.32* | -0.03 | 0.39* | (0.89) |  |
| Note. *N* = 227-230 (first-generation college student had only 227 observations); Cronbach’s alpha is on the diagonal in the parentheses for psychometric variables; Gender was coded with 1 = female, 2 = male, Academic degree was coded as 1 = yes, 2 = no; First-gen = first-generation college student (1 = yes, 0 = no); PLE = past leadership experience (1 = yes, 0 = no); AFF-MTL = affective-identity motivation to lead; SN-MTL = social normative motivation to lead; NC-MTL = noncalculative motivation to lead; PS-MTL = prosocial motivation to lead. *p < .05 | | | | | | | | | | | | | | | |

**Table 4**

*Results of Regression Analyses for Leadership Effectiveness (Sample 3)*

|  | (1) | (2) | (3) | (4) | (5) |
| --- | --- | --- | --- | --- | --- |
| VARIABLES | Leader Effectiveness | Leader Effectiveness | Leader Effectiveness | Leader Effectiveness | Leader Effectiveness |
| Intercept | 0.15 | 0.21 | 0.21 | 0.21 | 0.14 |
|  | (0.20) | (0.18) | (0.18) | (0.18) | (0.18) |
| Gender | -0.17 | -0.16 | -0.16 | -0.16 | -0.11 |
|  | (0.11) | (0.10) | (0.10) | (0.10) | (0.10) |
| Age | 0.08 | 0.07 | 0.07 | 0.07 | 0.07 |
|  | (0.06) | (0.06) | (0.06) | (0.06) | (0.06) |
| Academic Degree | -0.05 | -0.03 | -0.03 | -0.03 | -0.02 |
|  | (0.15) | (0.14) | (0.14) | (0.14) | (0.13) |
| PLE | 0.14 | -0.01 | -0.01 | -0.01 | 0.02 |
|  | (0.12) | (0.11) | (0.11) | (0.11) | (0.11) |
| First-gen | 0.07 | 0.06 | 0.06 | 0.06 | 0.04 |
|  | (0.11) | (0.10) | (0.10) | (0.10) | (0.10) |
| Leadership Self-Efficacy | 0.33*** | 0.27*** | 0.26*** | 0.26*** | 0.24*** |
|  | (0.06) | (0.06) | (0.06) | (0.06) | (0.06) |
| Leadership Aspiration | 0.35*** | 0.16** | 0.15** | 0.15** | 0.12* |
|  | (0.06) | (0.06) | (0.06) | (0.07) | (0.06) |
| AFF-MTL |  | 0.42*** | 0.42*** | 0.42*** | 0.39*** |
|  |  | (0.06) | (0.07) | (0.07) | (0.06) |
| SN-MTL |  |  | 0.05 | 0.05 | -0.00 |
|  |  |  | (0.06) | (0.06) | (0.06) |
| NC-MTL |  |  |  | -0.01 | 0.06 |
|  |  |  |  | (0.05) | (0.05) |
| PS-MTL |  |  |  |  | 0.19*** |
|  |  |  |  |  | (0.06) |
| R² | 0.33 | 0.44 | 0.45 | 0.45 | 0.47 |
| ΔR² |  | 0.11 | 0.01 | 0.00 | 0.02 |
| R² Adjusted | 0.31 | 0.42 | 0.42. | 0.42 | 0.44 |
| F | 15.62*** | 21.72*** | 19.36*** | 17.34*** | 17.38*** |
| Note. *N* = 227; robust standard errors in parentheses; The baseline for ΔR² is established by Model 1. Gender was coded with 1 = female, 2 = male Academic degree was coded as 1 = yes, 2 = no; PLE = past leadership experience (1 = yes, 0 = no); First-gen = first-generation college student (1 = yes, 0 = no); AFF-MTL = affective-identity motivation to lead; SN-MTL = social normative motivation to lead; NC-MTL = noncalculative motivation to lead; PS-MTL = prosocial motivation to lead. *** p<0.001, ** p<0.01, * p<0.5 | | | | | |

**Appendix B. Female Leadership Strength Awareness (FLSA)**

**Table 1**

*Descriptive Statistics and Correlations (Sample 2)*

| Variables | Mean | SD | (1) | (2) | (3) | (4) | (5) | (6) | (7) | (8) | (9) | (10) | (11) | (12) | (13) |
| --- | --- | --- | --- | --- | --- | --- | --- | --- | --- | --- | --- | --- | --- | --- | --- |
| (1) Gender | 0.64 | 0.48 | - |  |  |  |  |  |  |  |  |  |  |  |  |
| (2) Age | 30.4 | 13.1 | 0.04 | - |  |  |  |  |  |  |  |  |  |  |  |
| (3)Academic degree | 0.40 | 0.49 | 0.00 | 0.47* | - |  |  |  |  |  |  |  |  |  |  |
| (4) PLE | 0.69 | 0.46 | 0.03 | 0.25* | 0.11 | - |  |  |  |  |  |  |  |  |  |
| (5) Leadership Self-Efficacy | 4.37 | 0.44 | 0.03 | -0.04 | -0.12 | 0.09 | (0.91) |  |  |  |  |  |  |  |  |
| (6) Leadership Aspiration | 4.95 | 0.83 | 0.09 | -0.08 | -0.08 | 0.22* | 0.35* | (0.83) |  |  |  |  |  |  |  |
| (7) AGI | 4.13 | 0.86 | -0.20* | 0.22* | 0.01 | 0.05 | -0.08 | -0.00 | (0.82) |  |  |  |  |  |  |
| (8) SSRM | 0.28 | 0.45 | -0.20* | -0.09 | 0.04 | 0.19* | 0.10 | 0.06 | -0.03 | - |  |  |  |  |  |
| (9) FLSA | 4.62 | 1.10 | 0.40* | -0.09 | -0.14 | 0.05 | 0.15* | 0.17* | -0.26* | 0.03 | (0.81) |  |  |  |  |
| (10) AFF-MTL | 4.76 | 1.04 | 0.03 | 0.07 | -0.04 | 0.33* | 0.28* | 0.43* | -0.00 | 0.11 | 0.02 | (0.89) |  |  |  |
| (11) SN-MTL | 4.40 | 0.91 | 0.00 | -0.07 | -0.03 | 0.20* | 0.28* | 0.35* | -0.02 | 0.23* | 0.16* | 0.33* | (0.73) |  |  |
| (12) NC-MTL | 4.45 | 0.83 | 0.12 | 0.26* | 0.13 | -0.04 | 0.01 | -0.05 | -0.04 | -0.15* | -0.03 | 0.02 | -0.10 | (0.85) |  |
| (13) PS-MTL | 5.93 | 0.72 | 0.14* | -0.15* | -0.06 | 0.17* | 0.29* | 0.40* | -0.13 | 0.07 | 0.26* | 0.29* | 0.37* | 0.13 | (0.85) |
| N = 200-212 (Academic degree had only 200 observations). Cronbach’s alpha is on the diagonal in the parentheses for psychometric variables. Gender was coded as 1 = female, 0 = male; Academic degree was coded as 1 = yes, 2 = no; PLE = past leadership experience (1 = yes, 0 = no); AGI = awareness of gender inequality; SSRM = same-sex role model; FLSA = female leadership strength awareness; AFF-MTL = affective-identity motivation to lead; SN-MTL = social normative motivation to lead; NC-MTL = noncalculative motivation to lead; PS-MTL = prosocial motivation to lead | | | | | | | | | | | | | | | |

**Table 2**

*Average-Variance-Extracted, Square-Root Correlations, and HTMT (Sample 2)*

| Variables | (1) | (2) | (3) | (4) | (5) |
| --- | --- | --- | --- | --- | --- |
| (1) Female Leadership Strength Awareness | *0.52* | 0.04 | 0.13 | 0.40 | 0.02 |
| (2) Leadership Self-Efficacy | 0.00 | *0.69* | 0.72 | 0.02 | 0.11 |
| (3) Leadership Aspiration | 0.02 | 0.52 | *0.59* | 0.03 | 0.05 |
| (4) Awareness of Gender Inequality | 0.16 | 0.00 | 0.00 | *0.58* | 0.06 |
| (5) Same-Sex Role Model | 0.00 | 0.01 | 0.00 | 0.00 | *1.00* |
| N = 212; Coefficients below the diagonal indicate squared correlations. Coefficients at the diagonal indicate the AVE of each variable. Coefficients above the diagonal indicate the HTMT values. | | | | | |

**Table 3**

*Descriptive Statistics and Correlations (Sample 5)*

| Variables | Mean | SD | (1) | (2) | (3) | (4) | (5) | (6) | (8) | (9) | (10) | (11) | (12) | (13) | (14) |
| --- | --- | --- | --- | --- | --- | --- | --- | --- | --- | --- | --- | --- | --- | --- | --- |
| (1) Age | 39.9 | 12.18 | - |  |  |  |  |  |  |  |  |  |  |  |  |
| (2) Academic degree | 5.59 | 1.16 | -0.11* | - |  |  |  |  |  |  |  |  |  |  |  |
| (3) First-gen | 0.58 | 0.49 | 0.08 | -0.14* | - |  |  |  |  |  |  |  |  |  |  |
| (4) PLE | 0.76 | 0.43 | 0.30* | -0.04 | -0.00 | - |  |  |  |  |  |  |  |  |  |
| (5) Leadership Aspiration | 5.31 | 1.38 | 0.04 | 0.02 | -0.01 | 0.21* | (0.84) |  |  |  |  |  |  |  |  |
| (6) Leadership Self-Efficacy | 5.67 | 1.32 | 0.19* | -0.04 | 0.04 | 0.33* | 0.55* | (0.80) |  |  |  |  |  |  |  |
| (7) SSRM | 0.39 | 0.49 | 0.03 | 0.09* | -0.34* | 0.08 | 0.01 | -0.01 | - |  |  |  |  |  |  |
| (8) FLSA | 5.14 | 1.12 | 0.03 | -0.03 | 0.08* | 0.12* | 0.22* | 0.19* | 0.02 | (0.79) |  |  |  |  |  |
| (9) AFF-MTL | 5.16 | 1.32 | 0.20* | -0.03 | 0.02 | 0.38* | 0.64* | 0.67* | 0.04 | 0.18* | (0.90) |  |  |  |  |
| (10) SN-MTL | 3.86 | 1.32 | 0.05 | 0.01 | -0.05 | 0.10* | 0.30* | 0.16* | 0.07 | 0.13* | 0.22* | (0.75) |  |  |  |
| (11) NC-MTL | 5.09 | 1.57 | 0.05 | -0.03 | -0.01 | 0.06 | 0.08 | 0.12* | 0.03 | -0.00 | 0.11* | 0.15* | (0.88) |  |  |
| (12) PS-MTL | 6.40 | 0.72 | -0.01 | -0.05 | 0.09* | 0.15* | 0.36* | 0.32* | -0.06 | 0.23* | 0.34* | 0.21* | 0.20* | (0.81) |  |
| (13) Leadership Effectiveness | 5.01 | 1.12 | 0.23* | -0.03 | 0.08* | 0.30* | 0.50* | 0.62* | -0.03 | 0.31* | 0.57* | 0.16* | 0.02 | 0.23* | (0.90) |
| N = 551. Cronbach’s alpha is on the diagonal in the parentheses for psychometric variables. Academic degree was coded as 1 = yes, 2 = no; First-gen = first-generation college student (1 = yes, 0 = no); PLE = past leadership experience (1 = yes, 0 = no); AGI = awareness of gender inequality; SSRM = same-sex role model; FLSA = female leadership strength awareness; AFF-MTL = affective-identity motivation to lead; SN-MTL = social normative motivation to lead; NC-MTL = noncalculative motivation to lead; PS-MTL = prosocial motivation to lead. | | | | | | | | | | | | | | | |

**Table 4**

*Results of Regression Analyses for MTL and Leadership Effectiveness (Sample 5)*

|  | (1) | (2) | (3) | (4) | (5) | (6) | (7) | (8) | (9) | (10) |
| --- | --- | --- | --- | --- | --- | --- | --- | --- | --- | --- |
| VARIABLES | AFF-MTL | SN-MTL | NC-MTL | PS-MTL | LE | AFF-MTL | SN-MTL | NC-MTL | PS-MTL | LE |
|  |  |  |  |  |  |  |  |  |  |  |
| Intercept | -0.67* | -0.21 | 0.06 | -0.16 | -0.57* | -0.63** | -0.17 | 0.06 | -0.10 | -0.49* |
|  | (0.23) | (0.24) | (0.24) | (0.24) | (0.23) | (0.22) | (0.24) | (0.24) | (0.24) | (0.22) |
| Age | 0.09** | 0.03 | 0.03 | -0.07 | 0.15** | 0.09* | 0.03 | 0.03 | -0.06 | 0.15*** |
|  | (0.04) | (0.05) | (0.05) | (0.05) | (0.04) | (0.04) | (0.045) | (0.05) | (0.04) | (0.04) |
| Academic degree | 0.00 | 0.01 | -0.03 | -0.03 | 0.01 | 0.00 | 0.01 | -0.03 | -0.03 | 0.02 |
|  | (0.04) | (0.04) | (0.04) | (0.04) | (0.04) | (0.03) | (0.04) | (0.04) | (0.04) | (0.03) |
| First-gen | 0.03 | -0.08 | -0.02 | 0.14 | 0.13 | 0.01 | -0.11 | -0.01 | 0.10 | 0.07 |
|  | (0.09) | (0.09) | (0.09) | (0.09) | (0.09) | (0.09) | (0.09) | (0.09) | (0.09) | (0.08) |
| PLE | 0.83*** | 0.21* | 0.11 | 0.40*** | 0.61*** | 0.79*** | 0.18 | 0.11 | 0.34** | 0.53*** |
|  | (0.10) | (0.11) | (0.11) | (0.10) | (0.10) | (0.10) | (0.11) | (0.11) | (0.10) | (0.10) |
| SSRM | 0.03 | 0.09 | 0.06 | -0.10 | -0.07 | 0.02 | 0.08 | 0.06 | -0.12 | -0.10 |
|  | (0.09) | (0.09) | (0.09) | (0.09) | (0.09) | (0.09) | (0.09) | (0.09) | (0.09) | (0.08) |
| FLSA |  |  |  |  |  | 0.14*** | 0.13** | -0.01 | 0.21*** | 0.28*** |
|  |  |  |  |  |  | (0.040) | (0.04) | (0.04) | (0.04) | (0.04) |
|  |  |  |  |  |  |  |  |  |  |  |
| R² | 0.15 | 0.02 | 0.01 | 0.04 | 0.12 | 0.17 | 0.03 | 0.01 | 0.08 | 0.19 |
| R² Adjusted | 0.14 | 0.01 | -0.01 | 0.03 | 0.11 | 0.16 | 0.02 | -0.01 | 0.07 | 0.18 |
| F | 19.76*** | 1.74 | 0.68 | 4.16** | 14.49*** | 18.94*** | 2.97** | 0.57 | 7.72*** | 21.67*** |
| N = 551; robust standard errors in parentheses; predictors were z-standardized for comparability; Academic degree was coded as 1 = yes, 2 = no; First-gen = first-generation college student (1 = yes, 0 = no); PLE = past leadership experience (1 = yes, 0 = no); AGI = awareness of gender inequality; SSRM = same-sex role model; FLSA = female leadership strength awareness; AFF-MTL = affective-identity motivation to lead; SN-MTL = social normative motivation to lead; NC-MTL = noncalculative motivation to lead; PS-MTL = prosocial motivation to lead; LE = leadership effectiveness. *** p<0.001, ** p<0.01, * p<0.5 | | | | | | | | | | |
